# Chronic administration of XBD173 ameliorates cognitive deficits and neuropathology via 18 kDa translocator protein (TSPO) in a mouse model of Alzheimer’s disease

**DOI:** 10.1038/s41398-023-02630-z

**Published:** 2023-10-27

**Authors:** Arpit Kumar Pradhan, Tatjana Neumüller, Claudia Klug, Severin Fuchs, Martin Schlegel, Markus Ballmann, Katharina Johanna Tartler, Antoine Pianos, Maria Sanchez Garcia, Philippe Liere, Michael Schumacher, Matthias Kreuzer, Rainer Rupprecht, Gerhard Rammes

**Affiliations:** 1 Klinik für Anaesthesiologie und Intensivmedizin der Technischen Universität München, Klinikum rechts der Isar, Munich, Germany; 2 Graduate School of Systemic Neurosciences, Ludwig-Maximilians-Universität München, Martinsried, Germany; 3 U1195 Inserm and University Paris-Saclay, 80 rue du Général Leclerc, Le Kremlin-Bicêtre, 94276 France; 4 Department of Psychiatry and Psychotherapy, University Regensburg, Regensburg, Germany

**Keywords:** Learning and memory, Diseases, Physiology

## Abstract

Alzheimer’s disease (AD) is characterized by the accumulation of β-amyloid peptide (Aβ). It affects cognition and leads to memory impairment. The mitochondrial translocator protein (TSPO) plays an essential role in maintaining mitochondrial homeostasis and has been implicated in several neuronal disorders or neuronal injuries. Ligands targeting the mitochondrial translocator protein (18 kDa), promote neurosteroidogenesis and may be neuroprotective. To study whether the TSPO ligand XBD173 may exert early neuroprotective effects in AD pathology we investigated the impact of XBD173 on amyloid toxicity and neuroplasticity in mouse models of AD. We show that XBD173 (emapunil), via neurosteroid-mediated signaling and delta subunit-containing GABA_A_ receptors, prevents the neurotoxic effect of Aβ on long-term potentiation (CA1-LTP) in the hippocampus and prevents the loss of spines. Chronic but not acute administration of XBD173 ameliorates spatial learning deficits in transgenic AD mice with arctic mutation (ArcAβ). The heterozygous TSPO-knockout crossed with the transgenic arctic mutation model of AD mice (het TSPOKO X ArcAβ) treated with XBD173 does not show this improvement in spatial learning suggesting TSPO is needed for procognitive effects of XBD173. The neuroprotective profile of XBD173 in AD pathology is further supported by a reduction in plaques and soluble Aβ levels in the cortex, increased synthesis of neurosteroids, rescued spine density, reduction of complement protein C1q deposits, and reduced astrocytic phagocytosis of functional synapses both in the hippocampus and cortex. Our findings suggest that XBD173 may exert therapeutic effects via TSPO in a mouse model of AD.

## Introduction

Alzheimer’s disease (AD) is characterized by memory impairment and a progressive cognitive decline which has been attributed to the accumulation of Aβ in the brain. AD pathophysiology is largely reflected by the extracellular aggregation of Aβ peptide, hyperphosphorylated tau aggregates, and reactive gliosis [1]. Increased levels of pro-inflammatory cytokines such as interleukin-1β (IL-1β) have been shown to promote neurotoxicity induced by N-methyl-D-aspartate (NMDA) receptors, which is further amplified by the presence of Aβ [2, 3]. Aβ, a product of proteolytic cleavage (by β-secretases and γ-secretases) of amyloid precursor protein (APP) via the amyloidogenic pathway, may accumulate in AD brains both as a consequence of excessive production and impaired clearance [4]. The Aβ_1–42_ peptide has a higher propensity to aggregate and is highly enriched in amyloid deposits. Meanwhile, numerous studies have reported that soluble Aβ_1-42_ oligomers impair cognitive function and inhibit long-term potentiation (LTP), a cellular correlate for learning and memory [5–8]. Ample evidence suggests that Aβ_1-42_ pathologic effects are mediated by alterations in glutamatergic signaling, specifically NMDA receptors and possibly metabotropic glutamate receptors [9]. A fundamental yet unresolved question in the field of AD pathology is whether the Aβ assemblies exert varying effects on different neurons and synapses. This is crucial for predicting the effects of Aβ on the output of the neuronal circuits and the network activity [10, 11]. For instance, if Aβ impairs the synaptic function of inhibitory interneurons more than that of excitatory principal cells, it would likely cause dis-inhibition and over-excitation, rather than suppression, at the network level. GABAergic impairments have been frequently associated with AD pathophysiology. Neuronal network dysfunction such as widespread epileptiform activity and changes in neuronal synchronization have been found in patients and animal models of AD [11–13]. The impairment of GABAergic function in AD is frequently attributed to these seizures [14]. Additionally, increased neuronal activity also elevates APP processing, and Aβ production and modulates synaptic function [15]. Targeting the GABAergic activity, therefore, could present a suitable therapeutic action by affecting the excitatory tone as well as the increased amyloidogenic processing of APP in AD pathology.

The 18 kDa TSPO protein is known to orchestrate several important functions including cholesterol transfer into mitochondria, TSPO ligands-induced neurosteroidogenesis, mitochondrial homeostasis, gliosis, and apoptosis [16, 17]. Contrary to benzodiazepines which are positive allosteric modulators of GABA_A_ receptors, neurosteroids act through a different site than that targeted by benzodiazepines. For example, the δ-subunit of GABA_A_ receptor is considered to be highly sensitive to neurosteroids but not to benzodiazepines [18]. This potentially explains the positive effects of synaptic modulation by neurosteroids because of a more favorable side-effect profile.

TSPO ligands are neuroprotective in a range of neurological and psychiatric disorders [16, 19]. Prior studies investigating the role of TSPO in the pathophysiology of Alzheimer’s disease have yielded conflicting outcomes. The study by Ceyzériat et al. shows that TSPO plays a crucial role in the pathophysiology of AD, and knockout of TSPO ameliorates Tau-induced cognitive dysfunction and exhibits delayed and reduced amyloid pathology [20]. On the other hand, two recent studies reported that TSPO deficiency accelerated amyloid pathology and impaired Aβ phagocytosis [21, 22]. Previously, TSPO ligands such as Ro5-4864 and PK11195 have demonstrated the ability to confer neuroprotective advantages in terms of behavior and pathophysiology in mouse models of AD [23–25]. Moreover, the administration of Ro5-4864 has been shown to elevate levels of brain testosterone and progesterone in 3xTgAD mice [23]. However, the recent findings from Shi et al., suggest that diazepam via TSPO alters synaptic plasticity [26]. Additionally, both the ligands Ro5-4864 and PK11195 have been shown to alter the locomotor and exploratory behavior as well as affect band-specific changes in EEG [27]. As such, it remains to be resolved whether TSPO ligands exert beneficial or detrimental effects on neuroplasticity. Despite these conflicting studies, TSPO’s significant involvement in mitochondrial homeostasis, energy balance, steroidogenesis, gliosis, and apoptosis renders it a compelling target, particularly in pathological conditions like AD.

In this study, we asked whether XBD173, a TSPO ligand known to exert anxiolytic effect both in rodents and in humans [16, 28], may confer neuroprotective benefits in a murine model of AD. We show that XBD173 provides comprehensive neuroprotective benefits both ex vivo as well as in vivo in AD transgenic mouse model. These are mediated by a TSPO-dependent regulation of neurosteroids, which in turn affects the activity of the GABA_A_ receptor containing delta subunit. As such, our study demonstrates that the TSPO ligand XBD173 provides cognitive benefits by influencing multiple pathways in AD.

## Materials and methods

### Animals

All animal study protocols were in accordance with the German law on animal experimentation and were approved by the animal care committee (Technical University Munich, Munich, Germany) and the experiments performed confirmed the institutional regulatory standards. Mice (maximum of 6 mice per cage) were caged in a climate-controlled room (23 ± 0.5 °C) with 12 h of light and darkness with an *ad libitum* supply of food and water. All the acute slice experiments involved mice of both sexes which were 8–12 weeks old. Animals involved in the behavioral testing were housed under similar conditions except for 2 weeks before the water cross maze behavioral testing where mice were housed in a reverse light-dark condition (12-h light and darkness) cabinet maintained at a temperature of 23 ± 0.5 °C. Both male and female animals at the age of 8–9 months were given either chronic or acute treatment before the behavioral experiment.

Wild-type (C57BL/6, WT) mice were obtained from Charles River (Italy) and ArcAβ (APP E693G) mice were obtained from CALCO (Italy) [29]. The transgenic mice carrying this arctic mutation (ArcAβ) present a point mutation where glutamic acid (E) has been replaced by site-directed mutagenesis with glycine (G). The TSPOKO mice were bred by our group in Munich and had a global knockout of TSPO. The TSPOKO mice were generated in accordance with the previously established protocol [30]. TSPOKO X ArcAβ mice were generated by crossing stable lines of TSPOKO and TSPOKO X ArcAβ mice. The GABA δ knock-out (δ-KO) (line B6.129-Gabrdtm1Geh/J) mouse line was bred by our group in Munich (Germany) in a way similar to the Jackson Laboratory (Jax stock 003725). As a result of the KO mutation, the δ subunit of the GABA_A_ receptor is absent in these animals. The absence of the δ subunit in the GABA_A_ receptor of these mice leads to attenuated sensitivity to neurosteroids as described previously by Mihalek et al. (1999) [31]. Genotyping was done in each case to confirm the respective transgenic lines.

### Preparation of oligomeric Aβ and test compounds

Aβ_1-42_ (order number H-1368; Bachem, Bubendorf, Switzerland) and Aβ_1-40_ (Product Number: 4014442; Bachem, Bubendorf, Switzerland) were suspended in 100% hexafluoroisopropanol (HFIP, Sigma Aldrich) to a concentration of about 1 mg/400 µL HFIP and shaken at 37 °C for 1.5 h. Aliquots of 100 µg Aβ_1-42_ and Aβ_1-40_ were made per tube. The HFIP was then evaporated by using a Speedvac for 30 min. After the tubes were completely dry, they were labeled and stored at −80 °C. The respective Aβ were dissolved in DMSO (Sigma Aldrich) to obtain a concentration of 100 µM. This solution was then diluted to a concentration of 50 nM Aβ_1-42_ and Aβ_1-40_ using Ringer’s solution.

Diazepam and XBD173 were dissolved in DMSO. For LTP recordings, XBD173 was dissolved in aCSF to obtain a 300 nM concentration whereas, for other ex vivo slice experiments, XBD 173 was dissolved to a 3 µM concentration. For chronic and acute treatment for behavioral experiments, XBD173 and diazepam were diluted in a 0.9% NaCl solution. Drug doses were 1 mg/kg for XBD173 and 1 mg/kg for diazepam. For chronic treatment, intraperitoneal (i.p.) injections of XBD173, diazepam, or vehicle were given every alternate day for 12 weeks starting from 8–9 months while for the acute treatment group, the treatment XBD173(1 mg/kg), and diazepam (1 mg/kg) was given once 3 days before starting of the behavioral tests. The vehicle group received only DMSO diluted in 0.9% NaCl solution at a similar concentration as with XBD173.

### Acute slice preparation

Mice were deeply anesthetized with isoflurane before decapitation. The mouse brain was rapidly placed in an ice-cold Ringer solution consisting (in mM) of 125 NaCl, 2.5 KCl, 25 NaHCO_3_, 0.5 CaCl_2_, 6 MgCl_2_, 25 D-glucose and 1.2 NaH_2_PO_4_, with a pH of 7.3 and continuously oxygenated with carbogen gas (95% O_2_/5% CO_2_). Sagittal hippocampal slices (350 µm thickness) were obtained from both hemispheres using a microtome (HM 650 V; Microm International, Germany). Slices were recovered for 30 min at 35 °C in a chamber submerged with artificial cerebrospinal fluid (aCSF) containing (in mM) 125 NaCl, 2.5 KCl, 25 NaHCO_3_, 2 CaCl_2_, 1 MgCl_2_, 25 D-glucose and 1.2 NaH_2_PO_4_, and constantly oxygenated with carbogen. The slices were then placed at room temperature for 1 h of recovery before transferring them into the recording chamber. The slices in the recording chamber were fixed using a platinum ring with two nylon threads while carbonated aCSF was steadily perfused at a flow rate of 5 ml min^−1^. All recordings were performed at room temperature (21–23 °C).

### CA1-LTP recordings

LTP recordings were performed as previously established [32]. In brief, we recorded field excitatory postsynaptic potentials (fEPSPs) using glass micropipettes (1–2 MΩ) filled with aCSF solution in the CA1 region of the hippocampus. fEPSPs were recorded in the hippocampal CA1 stratum radiatum induced by stimulation in the Schaffer collaterals-commissural pathway. fEPSPs were evoked via alternating test stimulation (50 µs, 5–20 V) delivered through bipolar electrodes (Hugo Sachs Elektronik-Harvard Apparatus, Germany; 50 µm tip diameter) placed on both sides of the recording pipette. In this way, the non-overlapping populations of fibers of the Schaffer collateral-associational commissural pathway were stimulated. The stimulation intensity was adapted to values evoking an fEPSP slope of around 25–30% of the maximum response, for baseline recording. LTP was induced after at least 20 min of recording a stable baseline by delivering a high-frequency stimulation (HFS) train (100 pulses at 100 Hz over 1 s) through one of the two stimulating electrodes. The independent stimulation with two electrodes allowed the recording of internal control in the same slice. Following the administration of HFS in the absence of any substance, the potentiation of the fEPSP slope was measured for 60 min after the tetanic stimulus, while maintaining the settings used for the baseline. Briefly, HFS was delivered from one of the electrodes in the absence of Aβ (Aβ_1-40_ or Aβ_1-42_), and potentiation of the responses was monitored for at least 60 min following tetanus. Aβ (Aβ_1-40_ or Aβ_1-42_) was then applied via the bath solution for 90 min, before inducing the LTP in the second input via the HFS train.

To test toxicity prevention, HFS was delivered from one of the electrodes in the presence of either XBD173, pregnenolone, allopregnanolone, or allotetrahydrodeoxycorticosterone (THDOC), and the potentiation of the responses was monitored for at least 60 min after tetanic stimulation. After that, Aβ (Aβ_1-40_ or Aβ_1-42_) was applied via bath solution for 90 min before inducing LTP in the second input via the HFS train. LTP inhibition or blockage was defined as the fEPSP slope being less than 120% of the prestimulation slope in the last 10 min after HFS. The fEPSPs were amplified (BA-2S, npi electronic, Germany), filtered (3 kHz), and digitized (9 kHz) using an interface board (ITC-16, Instrutech Corp., USA) and the WinLTP program. Because stimuli were alternately delivered to each input every 15 s, two signals from the same input were averaged to one for analysis and one data point per minute. WinLTP program was used to analyze the fEPSPs offline. The slope of the fEPSP was measured between 20% and 80% of the peak amplitude which was then normalized to the 20-min control baseline recording obtained before the induction of LTP.

### Spine imaging

We used Thy1-eGFP mice for the dendritic spine analysis of ex vivo experiments with Aβ_1-42_. After the recovery period of 1 h at room temperature, the slices were treated with XBD173 and Aβ_1-42_. After incubation with respective treatment, slices were collected, fixed with 4% PFA for 2 days, and cryoprotected with 30% sucrose for 3 days. A cryostat (CryoStar NX70, Thermo Fisher Scientific, Bremen, and Germany) was used to prepare slices at a 100 µm thickness (Bremen, Germany). The slices were transferred to slides and coverslipped after being washed with PBS. Images of apical oblique dendritic spines in the CA1 region of the hippocampus were captured with a confocal microscope (Leica SP8, Wetzlar, Germany) with a Z step size of 0.3 µm and a 60X/1.40 N.A. oil-immersion objective. 6–8 dendrites of 20–40 µm in length from pyramidal neurons were obtained from the stratum radiatum layer in the CA1 region per mouse and were analyzed with the IMARIS 9.7 for Neuroscientists (Oxford Instruments, bitplane). Reconstruction of dendrites and spines was also performed using the Filament tool from the IMARIS package before categorizing the spines into individual subtypes. Dendritic spines were categorized as thin, mushroom, and stubby subtypes, based on established criteria. The following parameters were applied to automatically categorize the spines in the IMARIS: Mushroom Spines: length (spine) <3 and max_width (head) > mean_width (neck) * 2; Long Thin Spines: mean width(head) >= mean_width(neck) and Stubby Spines: length(spine) <1. The spine density was defined as the number of spines per µM of the dendrite.

### Behavioral test

Mice were trained in the water cross maze (WCM) using a previously described hippocampus-dependent place learning protocol [33]. The WCM set up tests for spatial learning and memory, where mice learn to use spatial cues to find a hidden platform to escape the water. Briefly, a plus-shaped maze made out of clear transparent plexiglass with four identical arms (length and width 50 cm and 10 cm) was designated with cardinal directions (N-, S-, E-, and W- arms). The maze was filled with fresh tap water (22 °C ± 1 °C) up to a height of 11 cm and a platform of the height of 10 cm and surface area (8 × 8 cm) was placed in the W arm. The light conditions were kept at <15 lux. A divider was placed to block off the fourth arm so that the mice chose either the E or W arm for escape. The entire period of this behavioral experiment was divided into 3 phases, a. Training phase (for a consecutive 5-day period) b. Retest 1 (performed after a week from the last day of the training phase) c. Retest 2 (performed after a month from retest 1). Retest 1 and retest 2 were designed to measure the retention of memory from the training phase and to observe if the drug effect is short-lived or long-lived. Each day had 6 randomized trials per mouse where the starting arms were alternated in a pseudorandomized order to make sure the mouse did not get accustomed to body turn to find the platform. After every trial, the mice were kept under infrared light and allowed sufficient time for rest before the next trial. A maximum time of 30 s was allotted for each mouse to find the platform. In the event of the mouse not finding the platform, the experimenter gently guided the mouse to the platform (in such cases latency of 31 s was noted).

We used the following parameters to evaluate spatial learning and memory: 1. Escape Latency: time to reach the platform from the starting arm (averaged over 6 trials every day per mouse) 2. Accuracy: Represents the number of correct trials per day in which the mice directly swam to the correct arm containing the platform without returning to the starting arm or the false arm (with no platform) 3. Accurate Learners: Number of mice that performed at least 5 out of 6 trials accurately per day, expressed as a percentage of the total number of mice per group. Accuracy change or escape latency change from the training phase in retests was calculated by subtracting the performance in respective retests with the mean of day 4 and day 5 in the training phase.

### Methoxy staining

Each mouse was anesthetized in an isoflurane chamber with a 5% isoflurane concentration at a flow rate of 2 L/min and the brain was decapitated using a guillotine. The brain was quickly frozen in dry ice and stored at −80 °C until further use for biochemical analyses. One hemisphere of the brain was cut in the sagittal plane into 30 µm thick slices using a cryotome (CryoStar NX70, Thermofisher). These 30 µm slices were incubated with methoxy-X04 solution (0.004 mg/ml, Tocris Bioscience, 1:1 ethanol-PBS solution) at room temperature for 30 min. The unspecific dye was removed by washing it three times with a 1:1 Ethanol-PBS solution and two times with Milli-Q water, and the slices were then mounted on microscope slides with a fluorescent mounting medium (Dako, Germany). 3D image stacks were obtained on an epi-fluorescence microscope (Axio Imager.M2 with ApoTome.2, Jena, Zeiss, Germany). Tile scan mode was used to image the brain slices which also allows the automatic stitching of the different brain segments. The excitation wavelength for the methoxy staining was fixed at 405 nm, and the emitted light was collected from 410 to 585 nm. The area and number of plaques were counted after the removal of the background using Fiji (ImageJ).

### ELISA

The other hemisphere of the frozen mouse brain was cut coronally and segmented to the cortex and hippocampus. The mass of the hippocampus and the cortex in the Eppendorf were noted and 8 X mass cold 5 M guanidine HCl/50 mM Tris HCl was added to each sample. The samples were ground thoroughly using a pestle. The homogenate was mixed with a 3D shaker for 3 h at room temperature. The homogenates were then diluted in 10 volumes of ice-cold casein buffer consisting of: 0.25% casein, 0.05% sodium azide, 20 µg/ml aprotinin, 5 mmol/l EDTA, 10 µg/ml leupeptin in PBS (pH 8.0). This was then centrifuged at 12,000 × *g* for 20 min at 4 °C. The supernatant was decanted and stored on ice until it was used with the ELISA kit (Human Aß Amyloid [34] ELISA kit; Invitrogen KHB 3441). The ELISA protocol was performed per the manufacturer’s instructions. The absorbance was read at 450 nm (Tecan Sunrise; Tecan Trading AG, Switzerland) within 30 min after adding the stop solution, and the concentration was read from the standard curve.

### Flow cytometry

After pharmacological treatment with XBD173 and Aβ_1-42_, the hippocampus and cortex were separated for each slice. These were then transferred to a 1 ml digestion solution (activated at 37 °C for 5 min) consisting of ethylenediaminetetraacetic acid (EDTA) (2 mM), L-cysteine (5 mM), and papain (5 units/ml). The enzymatic digestion was performed for 15 min at 37 °C. After the dissociation of slices, the suspension was filtered through a 70 µm cell strainer to obtain a single-cell suspension. After rinsing the cell strainer with 2 ml PBS, the samples were centrifuged for 10 min at 600 × *g* at room temperature. The supernatant was discarded and the cell pellet was resuspended in 1 ml Fluorescence-activated cell sorting (FACS) buffer consisting of EDTA (1 mM), sodium azide (15 mM), and 1% w/v bovine serum albumin (BSA). Cell count and % viability per sample were checked via a hemocytometer. To prepare the cells for FACS analysis, they were spun down at 600 x g for 10 min at 4 °C before staining with fluorescent antibodies. The cell pellets were resuspended in a 50 µl blocking mix (49 µl FACS buffer + 1 µl anti-CD16/32 antibody) per sample and incubated in the dark for 5 min at 4 °C. The samples were then incubated in the dark at 4 °C for 30 min with an antibody full-staining mix (Table 1). After adding 100 µl of FACS buffer, the cells were centrifuged at 600 × *g* for 5 min at 4 °C. Afterwards, the pellets were resuspended in 150 µl of FACS fixation buffer and stored at 4 °C until the FACS analysis was performed. FACS analysis was performed by the BD LSRFortessa™ X-20 using the FACSDiva™ data acquisition software. Compensation was performed for each channel using compensation data measured previously with compensation beads. The events were then gated for microglia or the astrocytes. Supplementary Fig. 1A depicts the gating strategy. Antibodies used in the FACS analysis and the source are listed in Table 2.

**Table 1:** Antibody Full-staining mix.

Antibody target	Coupled fluorochrome	µl per sample
CD45	Brilliant Violet 510	1
CD11b	Brilliant Violet 785	1
F4/80	PerCP-Cy5.5	1
TMEM119	PE-Cy7	2
P2RY12	APC	2
CD163	SuperBright 702	2
CD80	Brilliant Violet 605	1
CX3CR1	Brilliant Violet 421	1
GFAP	DyLight 594	1
IL-1β	APC-Cy7	1
ALDH1L1	DyLight 680	1
MHCII	BUV395	1
C1q	Alexa Fluor™ Plus 488	1
TSPO	Alexa fluor® 555	1
		14

**Table 2:** Antibodies used in the study.

Antibodies	Catalog number	Manufacturer
TruStain FcX™ (anti-mouse CD16/32) Antibody	101320	BioLegend, San Diego (USA)
FITC anti-mouse CD45 Antibody	147709	BioLegend, San Diego (USA)
APC/Cyanine7 anti-mouse/human CD11b Antibody	101225	BioLegend, San Diego (USA)
PE anti-mouse CD163 Antibody	155307	BioLegend, San Diego (USA)
Brilliant Violet 605™ anti-mouse CD80 Antibody	104729	BioLegend, San Diego (USA)
PerCP/Cyanine5.5 anti-mouse F4/80 Antibody	123127	BioLegend, San Diego (USA)
APC anti-P2RY12 Antibody	848005	BioLegend, San Diego (USA)
Brilliant Violet 421™ anti-mouse CX3CR1 Antibody	149023	BioLegend, San Diego (USA)
Tmem119 Monoclonal Antibody (V3RT1GOsz), PE-Cyanine7	25-6119-80	ThermoFisher Invitrogen
Recombinant Anti-C1q antibody	ab182451	Abcam
Anti-PBR antibody	ab118913	Abcam
Goat anti-Rabbit IgG (H + L) Secondary Antibody, Alexa Fluor™ Plus 488	A32731	Thermo Fisher
Donkey Anti-Goat IgG H&L (Alexa Fluor® 555)	ab150130	Abcam
Iba1/AIF-1 (E4O4W) XP® Rabbit mAb	17198	Cell Signaling Technology, Frankfurt am Main (GER)

### Immunofluorescent staining

#### Synaptic engulfment measurement

Sagittal brain slices, 30 µm thick were used for immunofluorescent staining. The slide containing the tissue sections was fixated in 1:1 acetone isopropanol solution at room temperature for 20 min. The sections were then permeabilized with 0.3% triton-X in PBS containing 0.1% tween 20 (PBS/T). This was followed by a 3X wash with PBS/T. The sections were blocked with 10% v/v normal goat serum (NGS) for 1 h at room temperature. This was followed subsequently by a 3X wash with PBS/T. The sections were incubated overnight at 4 °C with primary antibody diluted to respective concentrations in PBS/T. The primary antibody used were GFAP (GA5) Mouse mAb (1:600; Cell Signaling Tech, 3670 S), Rabbit anti-C1q antibody (1:250; Abcam, ab182451) and Rabbit anti-Synaptophysin antibody (1:600; Abcam, ab52636). On the subsequent day, the sections were incubated with secondary antibodies Goat Anti-Mouse IgG H&L (Alexa Fluor® 647) (1:700; Abcam, ab150115) and Goat Anti-Rabbit IgG Fc (Alexa Fluor® 488) (1:200; Abcam, ab150089) for 2 h at room temperature. For nuclear staining, ProLong™ Glass Antifade Mountant with NucBlue™ Stain (Sigma Aldrich, product no. 57-50-1) was used. If nuclear staining was not necessary the slides were mounted with DAKO fluorescent mounting media (DAKO, s3023).

#### Plaque astrocyte interaction

The slides containing sagittal brain sections (30 µm) were fixed with 4% paraformaldehyde (PFA) for 15 min at room temperature. The permeabilization and incubation with the primary and secondary antibodies were similar as described earlier in synaptic engulfment measurement. The primary antibodies used were GFAP (GA5) Mouse mAb (1:600; Cell Signaling Tech, 3670 S) and rabbit anti-C1q antibody (1:250; Abcam, ab182451). The secondary antibody used were Goat Anti-Mouse IgG H&L (Alexa Fluor® 647) (1:700; Abcam, ab150115) and Donkey anti-Rabbit IgG (H + L) Highly Cross-Adsorbed Secondary Antibody, Alexa Fluor Plus 647 (Invitrogen, A32795). After the incubation with the secondary antibody, the brain sections were washed with 3X PBS/T followed by 3 × 1:1 Ethanol-PBS solution for 5 min each. The sections were then incubated in the methoxy-04 solution in the dark. The unspecific dye was removed by washing it three times with a 1:1 Ethanol-PBS solution and two times with Milli-Q water, and the slices were then mounted on microscope slides with DAKO fluorescent mounting medium (DAKO, Germany).

#### 3D reconstruction of astrocytes and synaptic pruning

The 3D reconstruction of the astrocytes and measurement of synaptic engulfment in the volume of the astrocyte was performed using IMARIS 9.7 (Oxford Instruments, Bitplane) using the protocol as described previously [35].

##### Imaging setup

Leica Confocal SP8.

**Lasers:** 499 nm (C1q, Synaptophysin Intensity: 5–10%), 405 nm (NucBlueTM, Intensity: 10%), and 653 nm (GFAP, Intensity: 1–2%), laser power depends on staining efficacy. The power output was fixed at 70% of the maximum output (1.5 mW).

**Gain (Master, analog):** 800–1000 V.

**Frame size:** 1024 × 1024 pixels.

**Pinhole**: 1 AU.

**Digital Offset:** 0 for 405 channel (PMT), (HyD doesn’t require a digital offset).

**Scanning speed:** 200 Hz (lines per second).

**Z-stack scanning:** System optimized (z-step size normally 0.3 μm), scanning from the top to bottom of the focused astrocyte. On average, we collect 30–40 stacks over 6–8 μm (*Z* axis).

**Bit Depth:** 8-bit, bidirectional scanning.

**Objective:** 63× oil (Leica Type F Immersion liquid ne23 = 1,5180, ve = 46). NA = 1.40.

**Selection of channels:** C1q (Alexa 488, Abcam, green, 499 nm laser).

Rabbit anti-Synaptophysin antibody (1:600; Abcam, ab52636)

GFAP (Alexa 647, Cell Signaling Technology, red, 653 nm laser).

NucBlue™ Stain (405 nm laser).

**Emission Wavelength:** For C1q and Synaptophysin (510–530 nm).

GFAP (663–693 nm).

NucBlue™ Stain (415–457 nm).

% ROI colocalization measures (proportionally) the amount of synaptic tag engulfed by the entire astrocyte (GFAP) volume. The engulfment analysis was performed to calculate the amount of C1q (eat-me tag) and synaptophysin (pre-synaptic marker) inside the whole volume of individually reconstructed astrocytes. The interaction of plaque with astrocytes was done in a similar method. Instead of individual astrocytes, all the astrocytes in the frame were volumetrically reconstructed and their interaction with the plaques was measured by the percentage of the entire astrocytic volume that interacts with amyloid plaques. The astrocytes that were partially reconstructed or incorrectly rendered were removed manually. On a general note, the astrocyte within a 30 µm radius from the amyloid plaques was considered eligible for the plaque astrocyte interaction. The percentage interaction between C1q and plaque was done using “automatic thresholding” coloc analysis from the IMARIS package.

### Golgi-Cox staining of spines

Animals that went through the chronic or acute treatment for the water cross maze were decapitated using a guillotine and brains were sagitally sectioned to 250 µm hippocampal slices in the vibratome. The slices were recovered for 30 min at 35 °C in a chamber submerged with artificial cerebrospinal fluid (aCSF) and then at room temperature for an hour. The FD Rapid GolgiStain Kit (FD NeuroTechnologies) was used for Golgi–Cox staining. The staining was performed following the manufacturer’s instructions. The brain slices were incubated in an impregnation solution for four days. Golgi–Cox-stained pyramidal neurons of layer V were imaged for apical dendrites epi-fluorescence microscope (Axio Imager.M2 with ApoTome.2, Jena, Zeiss, Germany). The obtained Z-stacks were opened and analyzed in Fiji [36].

### Steroid profiling by gas chromatography coupled to tandem-mass spectrometry (GC-MS/MS)

A whole panel of steroids was identified and quantified simultaneously in individual tissues by a GC-MS/MS procedure fully validated in terms of accuracy, reproducibility, and linearity in the nervous tissue [37]. Briefly, steroids were extracted from the cortex and hippocampus with 10 volumes of MeOH. The following internal standards were added into the extracts for steroid quantification: 2 ng of ^13^C_3_-PROG for PROG, 2 ng of ^13^C_5_-5α-dihydroprogesterone (DHP) (CDN Isotopes, Sainte Foy la Grande, France) for 5α/β-DHP, 2 ng of epietiocholanolone (3β-hydroxy-5β-androstan-17-one, Steraloids, Newport, Rhode Island) for 5α/β-dihydrotestosterone (DHT), 3α/β5α/β-tetrahydrotestosterone (THT), pregnenolone (PREG), 3α/β5α/β-tetrahydroprogesterone (THPROG), 5α/β20α-THPROG, 3α/β5α/β20α/β-hexahydroprogesterone (HHPROG) and 3α/β5α/β-THDOC, (2 ng of 19-norPROG for 5α/β-dihydrodeoxycorticosterone (DHDOC), 10 ng of ^2^H_8_-corticosterone (B) for B and 5α/β-dihydrocorticosterone (DHB), 2 ng of ^13^C_3_-deoxycorticosterone (DOC) for DOC and 1 ng of ^13^C_3_-testosterone(T) for T. 2 ng of ^13^C_5_-20α-DHP (Isosciences) for the analysis of 20α/β-DHP.

Extracts were then purified and fractionated by solid-phase extraction with the recycling procedure by using a C18 cartridge (500 mg, International Sorbent Technology) (Liere et al, 2004). The unconjugated steroids-containing fraction was filtered and fractionated by the HPLC system composed of a WPS-3000SL analytical autosampler, an LPG-3400SD quaternary pump gradient coupled with an SR-3000 fraction collector (Thermoscientific, USA), and a Lichrosorb Diol column (25 cm, 4.6 mm, 5 µm) in a thermostated block at 30 °C. The column was equilibrated in a solvent system of 90% heptane and 10% of a mixture composed of heptane/isopropanol (85/15). Elution was performed at a flow rate of 1 ml/min, first with 90% heptane and 10% heptane/isopropanol (85/15) for 15 min, then with a linear gradient to 100% acetone in 2 min. This mobile phase was kept constant for 13 min. Two fractions were collected from the HPLC system: 5α/β-DHPROG were eluted in the first HPLC fraction (3–14 min) and were next silylated with 50 μl of a mixture N-methyl-N-trimethylsilyltrifluoroacetamide/ammonium iodide/dithioerythritol (1000:2:5) (vol/wt/wt) for 15 min at 70 °C. The second fraction (14–29 min) containing all other steroids was derivatized with 25 μl heptafluorobutyric anhydride (HFBA) and 25 μl anhydrous acetone for 1 h at 80 °C. Both fractions were dried under a stream of N2 and resuspended in heptane.

GC-MS/MS analysis of the purified and derivatized extracts was performed using an AI1310 autosampler, a Trace 1310 gas chromatograph (GC), and a TSQ8000 mass spectrometer (MS) (Thermoscientific, San Jose, CA). The injection was performed in the splitless mode at 280 °C (1 min of splitless time) and the temperature of the gas chromatograph oven was initially maintained at 80 °C for 1 min and ramped between 80 to 200 °C at 20 °C/min, then ramped to 300 °C at 5 °C/min and finally ramped to 350 °C at 30 °C/min. The helium carrier gas flow was maintained constant at 1 ml/min during the analysis. The transfer line and ionization chamber temperatures were 330 °C and 200 °C, respectively. Electron impact ionization was used for mass spectrometry with an ionization energy of 70 eV. Argon was used as the collision gas. GC-MS/MS signals were evaluated using a computer workstation using the software Excalibur®, release 3.0 (Thermoscientific, USA). Identification of steroids was supported by their retention time and according to two or three transitions. Quantification was performed according to the transition giving the more abundant signal. The range of the limit of detection was roughly 0.5–10 pg according to the steroid structure. The analytical protocol has been validated for all the targeted steroids using extracts of 200 mg of a pool of male mice brains. The evaluation included the limit of detection, linearity, accuracy, intra-assay, and inter-assay precision [37].

### Statistical analysis

All statistical analyses were performed with GraphPad Prism 6 (Version 6.01). The sample size used in the study was similar to previous publications using behavioral paradigms. The mice were randomly assigned to the treatment groups for both acute and chronic treatment and the sequence of behavioral tests were randomized. For the dendritic spine analysis, the experimenter was blinded to the mice genotype. The normality of the data sets was checked using the D’Agostino-Pearson and Shapiro–Wilk tests. Where the sample size was smaller, and normality detection was not possible, non-parametric testing was applied. In addition, data sets consisting of less than four samples were not considered for any statistical testing, instead, we used descriptive statistics to note the differences. For the CA1-LTP experiments, the sample size (n) is represented as x slices out of y animals i.e., *n* = 8/6 represents 8 slices obtained from 6 animals. The data sets are shown with the median and their respective interquartile range except for the escape latency and accuracy curves which are expressed as mean ± SD. The outliers in the sample sets were identified using the ROUT method with Q = 1%. For data sets not distributed normally, the significance of differences between experimental groups was accessed by the Kruskal–Wallis test followed by Dunn’s multiple comparisons *post hoc* test, and for normally distributed data sets one-way ANOVA followed by Bonferroni’s post-hoc analysis was used. The adjusted *p*-value after the multiple comparisons was calculated and is mentioned in the figure legends. Between two non-normally distributed experimental groups, the differences are analyzed by the Mann–Whitney *U* test. Each figure legend specifies the statistical test used along with the median and interquartile range for each data set. Statistical significance is indicated in the plots with an asterisk (*) when *p* < 0.05.

## Results

### XBD173 prevents the adverse synaptotoxic effects of Aβ_1-42_ on CA1-LTP in the hippocampus via TSPO

**Fig. 1:**
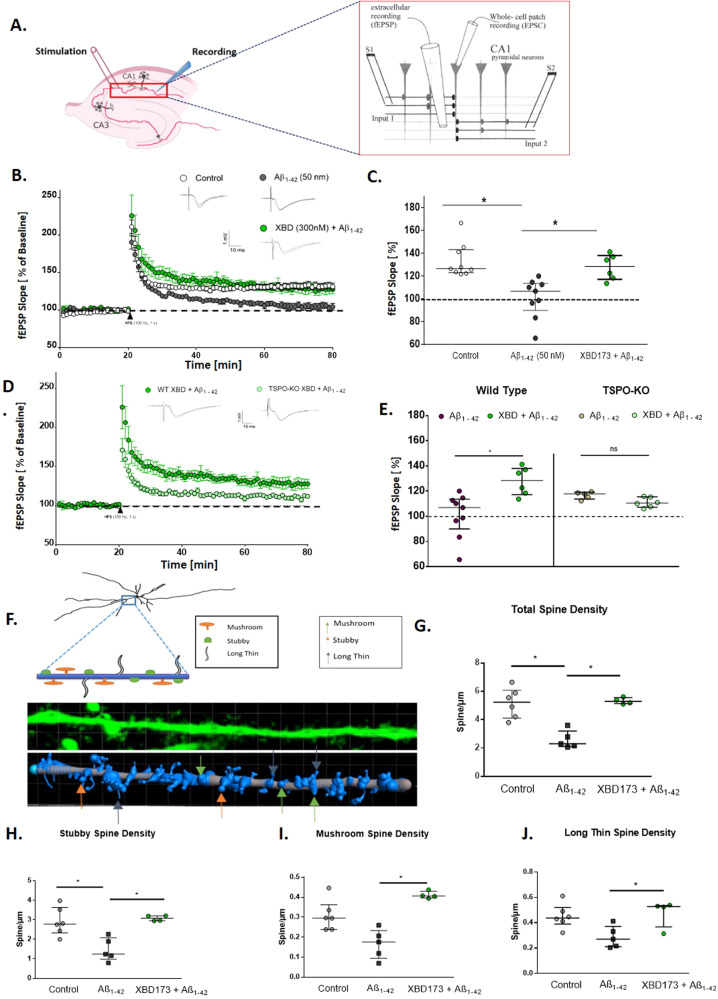
XBD173 via TSPO protein prevents the adverse synaptotoxic effects of Aβ_1-42_ on CA1-LTP in the hippocampus and prevents dendritic spine loss induced by Aβ_1-42_ oligomers. **A** Representative schematic for the positioning of electrodes to obtain CA1-LTP in the Schaffer collaterals pathway. The inset referring to the detailed positioning is adapted from [39]. **B** Normalized field excitatory postsynaptic potential (fEPSP) time course following a high-frequency stimulation (HFS) under control conditions, with 90 min Aβ_1-42_ exposure alone, and the simultaneous application of XBD173 (300 nM) and Aβ_1-42_ (50 nM) respectively. The insets on the top are representative traces for each treatment group. **C** Scatter dot plot summarizing the last 10 min (starting from 50 min to 60 min) after HFS for respective groups: Control (*n* = 10/10 [*n* = slices from animals]), Aβ_1-42_ (*n* = 9/9), XBD173 + Aβ_1-42_ (*n* = 6/6) (Kruskal–Wallis test with a Dunn’s multiple comparisons *post hoc* test; Control: 126.5 (123.0–143.3) % of baseline slope vs Aβ_1-42_: 106.9 (89.93–113.6) % of baseline slope, *p* = 0.0007; Aβ_1-42_ vs XBD173 + Aβ_1-42_: 128.5 (117.2–138.1) % of baseline slope, *p* = 0.0192). **D** Normalized field excitatory postsynaptic potential (fEPSP) time course following a high-frequency stimulation (HFS) in WT (XBD173 + Aβ_1-42_), and TSPO-KO (XBD173 + Aβ_1-42_). The insets on the top are representative traces for each treatment group. **E** Scatter dot plot summarizing the last 10 min (starting from 50 min to 60 min) after HFS for respective groups: WT Aβ_1-42_ (*n* = 9/9), WT XBD173 + Aβ_1-42_ (*n* = 6/6), TSPO-KO Aβ_1-42_ (*n* = 5/5) and TSPO-KO XBD173 + Aβ_1-42_ (*n* = 6/6) (Mann–Whitney *U* test: TSPO-KO Aβ_1-42_: 117.8 (113.7–119.1) % of baseline slope vs TSPO-KO XBD173 + Aβ_1-42_:110.5 (107.2–115.4) % of baseline slope, *p* = 0.0519; WT XBD173 + Aβ_1-42_ vs TSPO-KO XBD173 + Aβ_1-42_, *p* = 0.0087). **F** Schematic showing the different spine classifications. Below the schematics, the representative apical dendritic segments of the CA1 pyramidal neuron are shown. Dendrites and spines are reconstructed and classified using the Filament function in IMARIS Software. Different classes of spines are indicated with arrows: green: Mushroom, yellow: Stubby, and gray: Long thin spines. **G** Total spine density in different treatment groups: Control (*n* = 6), Aβ_1-42_ (*n* = 5), and XBD173 + Aβ_1-42_ (*n* = 4), (Kruskal–Wallis test with a Dunn’s multiple comparisons *post hoc* test; Control: 5.236 (4.108–6.080) spines/µm vs Aβ_1-42_: 2.302 (2.113–3.194) spines/µm, *p* = 0.0136; Aβ_1-42_ vs XBD173 + Aβ_1-42_: 5.297 (5.134–5.563) spines/µm, *p* = 0.0196) **H** Stubby spine density in different treatment groups: Control (*n* = 6), Aβ_1-42_ (*n* = 5), and XBD173 + Aβ_1-42_ (*n* = 4) (Kruskal–Wallis test with a Dunn’s multiple comparisons *post hoc* test; Control: 2.790 (2.332–3.639) spines/µm vs Aβ_1-42_: 1.244 (0.979–2.078) spines/µm, *p* = 0.0340; Aβ_1-42_ vs XBD173 + Aβ_1-42_: 3.086 (2.956–3.210) spines/µm, *p* = 0.0114). **I** Mushroom spine density in different treatment groups: Control (*n* = 6), Aβ_1-42_ (*n* = 5), and XBD173 + Aβ_1-42_ (*n* = 4) (Kruskal–Wallis test with a Dunn’s multiple comparisons *post hoc* test; Control: 0.2959 (0.2390–0.3629) spines/µm vs Aβ_1-42_: 0.1756 (0.0942-0.2332) spines/µm, *p* = 0.0897; Aβ_1-42_ vs XBD173 + Aβ_1-42_: 0.4063 (0.3966–0.4302) spines/µm, *p* = 0.0049). **J** Long-thin spine density in different treatment groups: Control (*n* = 6), Aβ_1-42_ (*n* = 5), and XBD173 + Aβ_1-42_ (*n* = 4) (Kruskal–Wallis test with a Dunn’s multiple comparisons *post hoc* test; Control: 0.4378 (0.3889–0.5203) spines/µm vs Aβ_1-42_: 0.2698 (0.2103–0.3712) spines/µm, *p* = 0.0846; Aβ_1-42_ vs XBD173 + Aβ_1-42_: 0.5272 (0.3662–0.5369) spines/µm, *p* = 0.0490). Data are represented as median with their respective interquartile range. *p < 0.05. ns not significant.

LTP can be induced by activating CA1 synapses in the hippocampus briefly with high-frequency electrical stimulation (100 pulses/100 Hz) [38] (Fig. 1A). The application of different Aβ oligomers e.g., Aβ_1-42_, 3NTyr10-Aβ, Aβ_1-40,_ and AβpE3 has been shown to impair CA1-LTP in acute hippocampal slices in a concentration-dependent manner [32]. Here, we tested whether the application of XBD173 prevents the synaptotoxic effects of Aβ_1-42_ and Aβ_1-40_ oligomers. In our experiments, we applied XBD173 concentration (300 nM), which still allows the induction of LTP. We used sagittal hippocampal slices and monitored the fEPSPs in the CA1 stratum radiatum of the hippocampus.

Low nanomolar concentration (50 nM) of both Aβ_1-42_ and Aβ_1-40_ inhibited CA1 LTP after 90 min of incubation in the bath solution (Fig. 1B, C, and Supplementary Fig. 2A, B). XBD173 (300 nM) preincubation for 60 min, prevented LTP impairments induced by Aβ_1-42_ oligomers (Fig. 1B, C). XBD173, however only partly rescues the LTP impairments resulting from the Aβ_1-40_ peptide (Supplementary Fig. 2A, B). To assess the role of TSPO protein in mediating the neuroprotective effect of XBD173, we also recorded the fEPSPs from the acute hippocampal slices obtained from the global TSPOKO mouse model. XBD173 fails to exert any neuroprotective effect in the transgenic TSPOKO mouse slices incubated with Aβ_1-42_ oligomers indicating a significant involvement of TSPO protein for the beneficial effect of XBD173 on ameliorating Aβ_1-42_-induced synaptic deficits (Fig. 1D, E).

### The TSPO ligand XBD173 promotes the synthesis of different neurosteroids which increases GABA_A_ receptor activity containing the GABA_A_ receptor delta subunit and provides neuroprotection against Aβ oligomers

**Fig. 2:**
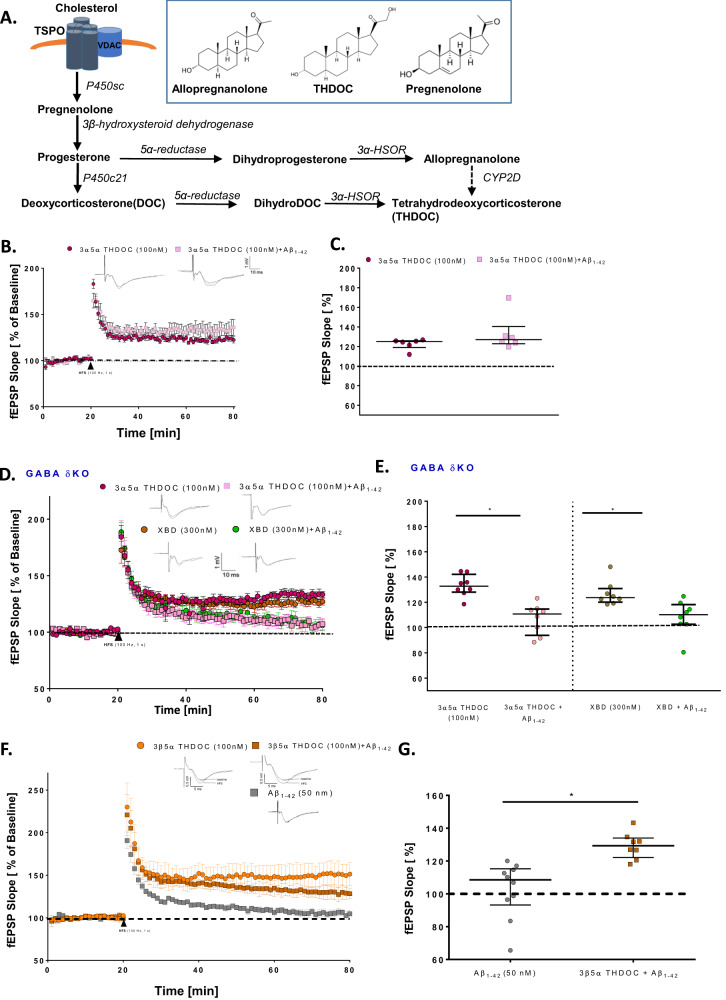
XBD173 promotes a TSPO-mediated synthesis of THDOC and other neurosteroids which enhances GABA_A_ receptor activity containing GABA delta subunit. **A**. Representative schematic of neurosteroid synthesis regulated by the TSPO protein. **B** Normalized field excitatory postsynaptic potential (fEPSP) time course following a high-frequency stimulation (HFS) under 3α5α-THDOC (100 nM) and 3α5α-THDOC + Aβ_1-42_ conditions in WT C57/Bl6 mice. The insets on the top are representative traces for each treatment group. **C** Scatter dot plot summarizing the last 10 min (starting from 50 min to 60 min) after HFS for respective groups: 3α5α-THDOC (*n* = 6/6 [*n* = slices from animals]) and 3α5α-THDOC + Aβ_1-42_ (*n* = 6/6) (Mann–Whitney *U* test: WT 3α5α-THDOC: 125.0 (119.0–126.0) % of baseline slope vs WT 3α5α-THDOC + Aβ_1-42_: 127.1 (122.9–140.5) % of baseline slope, *p* = 0.3874) **D**. Normalized field excitatory postsynaptic potential (fEPSP) time course following a high-frequency stimulation (HFS) under 3α5α-THDOC (100 nM), 3α5α-THDOC + Aβ_1-42_, XBD173 (300 nM) and XBD173 + Aβ_1-42_ conditions in transgenic GABA-δ KO mice. **E** Scatter dot plot summarizing the last 10 min (starting from 50 min to 60 min) after HFS for respective groups in GABA-δ KO mice: 3α5α-THDOC (100 nM) (*n* = 8/8), 3α5α-THDOC + Aβ_1-42_ (*n* = 8/8), XBD173 (300 nM) (*n* = 8/8) and XBD173 + Aβ_1-42_ (*n* = 8/8) (3α5α-THDOC: 132.7 (128.1–142.1) % of baseline slope vs 3α5α-THDOC + Aβ_1-42_: 110.8 (93.8–114.7) % of baseline slope, *p* = 0.0003; XBD: 123.7 (120.1–130.9) % of baseline slope vs XBD + Aβ_1-42_: 110.2 (102.7–118.2) % of baseline slope, *p* = 0.0070). **F** Normalized field excitatory postsynaptic potential (fEPSP) time course following a high-frequency stimulation (HFS) under 3β5α-THDOC (100 nM), Aβ_1-42_ (50 nM) and 3β5α-THDOC + Aβ_1-42_. **G** Scatter dot plot summarizing the last 10 min (starting from 50 min to 60 min) after HFS for respective groups in WT C57/Bl6 mice: Aβ_1-42_ (50 nM) (*n* = 10/10) and 3β5α-THDOC + Aβ_1-42_ (*n* = 8/8) (Mann–Whitney *U* test; Aβ_1-42_: 108.5 (93.18–115.3) % of baseline slope vs 3β5α-THDOC + Aβ_1-42_: 129.3 (122.1–134) % of baseline slope, *p* < 0.0001). Data are represented as median with their respective interquartile range. *p < 0.05. ns not significant.

Previously TSPO ligands have been attributed to increase neurosteroidogenesis [40] (Fig. 2A). Given the importance of TSPO dependency of XBD173, we investigated the role of different neurosteroids in preventing LTP impairment. TSPO ligands were previously shown to elevate the levels of the neurosteroids allopregnanolone and pregnenolone [41–43]. We started with different concentrations of neurosteroids and monitored the fEPSP response to see whether these neurosteroids could mimic the neuroprotective action of XBD173. Both pregnenolone (100 nM) and allopregnanolone (10 nM and 30 nM), were not able to prevent the LTP impairments from Aβ_1-42_ oligomers (Supplementary Figs. 3, 4). However, allopregnanolone (100 nM) was able to prevent the LTP impairment resulting from the Aβ_1-40_ peptide (Supplementary Fig. 5). In addition, 3α5α-THDOC (100 nM) was able to rescue the LTP impairment resulting from Aβ_1-42_ oligomers (Fig. 2B, C). Although both neurosteroids and benzodiazepines are positive allosteric modulators of GABA_A_ receptors they bind to different subunits. For instance, while benzodiazepines are sensitive to γ2 subunits, neurosteroids are sensitive to the δ subunit [34]. To test our hypothesis on neurosteroid dependence of XBD173-mediated effects on ameliorating the Aβ_1-42_ mediated synaptotoxicity, we used acute hippocampus slices from transgenic animals containing GABA-δ KO. 3α5α-THDOC (100 nM) was not able anymore to exert a beneficial action on the LTP in these slices. Additionally. XBD173 (300 nM) failed to exert the neuroprotective effect on GABA-δ KO hippocampal slices after treatment with the Aβ_1-42_ oligomers (Fig. 2D, E). These data suggest that the GABA_A_ receptor containing the δ subunit is required for the neuroprotective effects off the TSPO ligand XBD173. Similar to its 3α stereoisomer, 3β5α-THDOC also prevented the LTP impairment resulting from the incubation of Aβ_1-42_ oligomers (Fig. 2F, G). All these evidence suggests that XBD173 orchestrates the formation of several neurosteroids which play a crucial role in providing comprehensive neuroprotection against the Aβ derived pathophysiology. A summary graph showing the effects of different neurosteriods on Aβ mediated CA1-LTP impairment is shown in Supplementary Fig. 5C.

### XBD173 ex vivo treatment prevents dendritic spine loss resulting from Aβ_1-42_ oligomers

Dendritic spines are highly dynamic structures and critical for the function of neural circuits (Fig. 1F). An increase in spine density has been reported to accompany LTP and learning and memory-related mechanisms [44]. In previous experiments, we showed that Aβ reduces neuronal spines in hippocampal slices, and antagonizing GluN2b receptors reversed this effect [32]. Therefore, the application of XBD173 might be a promising approach to antagonize the over-activation of NMDA receptors on pyramidal cells. As such, in line with the beneficial effects of XBD173 against Aβ_1-42_ synaptotoxicity, we intended to unravel whether XBD173 can restore the detrimental effect of Aβ_1-42_ on spine density in hippocampal slices. XBD173 significantly prevented the elimination of spines induced by Aβ_1-42_ oligomers (Fig. 1G). Given the specific functionalities of different classes of spines, we also analyzed the spines according to their classification and quantified in detail stubby, long thin and mushroom spines. XBD173 significantly improved the stubby, mushroom, and long thin spine counts and prevented the synaptotoxic effect of Aβ_1-42_ oligomers on dendritic spines (Fig. 1H–J; Supplementary Fig. 6).

### Chronic but not acute administration of XBD173 ameliorates spatial learning deficits in AD mice via a TSPO-dependent pathway

**Fig. 3:**
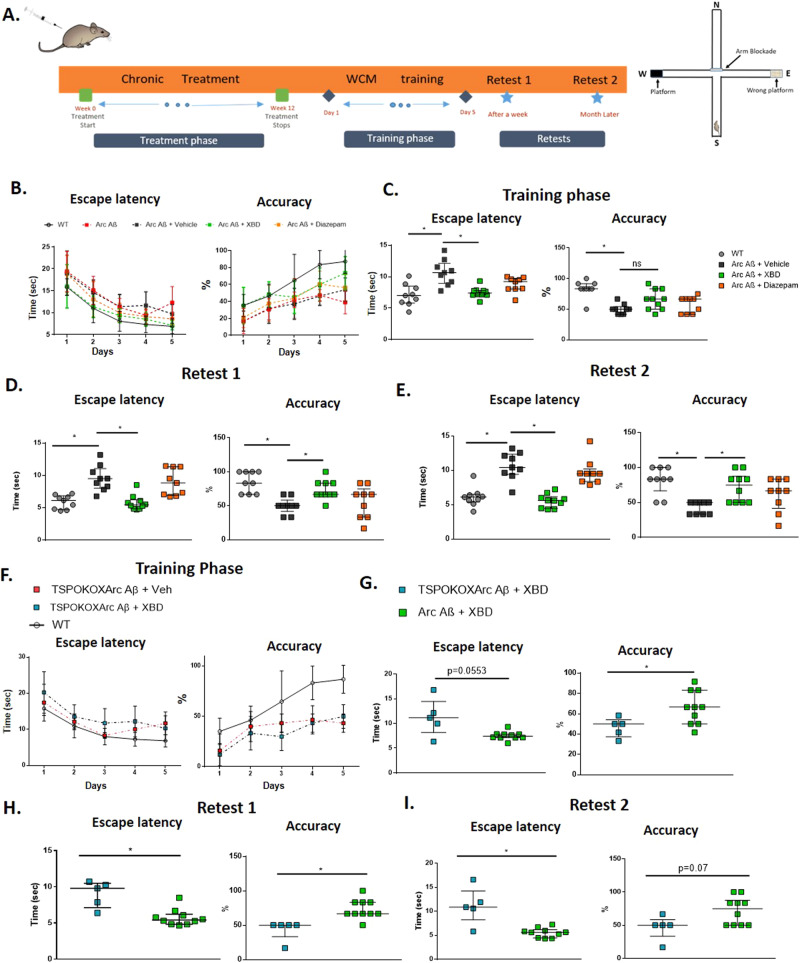
Chronic administration of XBD173 ameliorates cognitive deficits in AD mice via a TSPO-dependent pathway. **A** Schematic of chronic treatment schedule, training, and retests in water cross maze. **B** The escape latency and accuracy curves during the course of the 5-day training phase in the water cross maze. **C** Escape latency and accuracy comparison between the different treatment groups in the training phase. WT (*n* = 9), Arc Aβ + vehicle (*n* = 9), Arc Aβ + XBD (*n* = 10), and Arc Aβ + diazepam (*n* = 9) (Kruskal–Wallis test with a Dunn’s multiple comparisons *post hoc* test; WT: 7 (5.825–8.518) s vs Arc Aβ + vehicle: 10.66 (8.948–12.14) s, *p* = 0.0013; Arc Aβ + vehicle vs Arc Aβ + XBD: 7.420 (7.109–7.816) s, *p* = 0.0075; Arc Aβ + diazepam: 9.320 (8.073–9.758) s). Accuracy is expressed for each animal as the % of trials correctly performed (Kruskal–Wallis test with a Dunn’s multiple comparisons *post hoc* test; WT: 83.33 (83.33–91.67) % vs Arc Aβ + vehicle: 50 (41.67–54.17) %, *p* = 0.0004; Arc Aβ + vehicle vs Arc Aβ + XBD: 66.66 (50–83.33) %, *p* = 0.1155; Arc Aβ + diazepam: 66.66 (41.67–66.67) %). **D** Escape latency (Kruskal–Wallis test with a Dunn’s multiple comparisons *post hoc* test; WT: 6.113 (4.693–6.829) s vs Arc Aβ + vehicle: 9.477 (8.035–11.05) s, *p* = 0.0018; Arc Aβ + vehicle vs Arc Aβ + XBD: 5.430 (4.844–6.237) s, *p* = 0.0008; Arc Aβ + diazepam: 8.833 (6.931–11.34) s) and accuracy comparison (Kruskal–Wallis test with a Dunn’s multiple comparisons *post hoc* test; WT: 83.33 (66–100) % vs Arc Aβ + vehicle: 50 (41.67–58.33) %, *p* = 0.0003; Arc Aβ + vehicle vs Arc Aβ + XBD: 66.66 (50–66.66) %, *p* = 0.0145; Arc Aβ + diazepam: 66.66 (33.33–75) %) between the different treatment groups in the Retest 1 phase. WT (*n* = 9), Arc Aβ + vehicle (*n* = 9), Arc Aβ + XBD (*n* = 10) and Arc Aβ + diazepam (*n* = 9). **E** Escape latency (Kruskal–Wallis test with a Dunn’s multiple comparisons *post hoc* test; WT: 6.092 (5.375–6.498) s vs Arc Aβ + vehicle: 10.41 (9.426–12.35) s, *p* = 0.0007; Arc Aβ + vehicle vs Arc Aβ + XBD: 5.608 (4.444–6.166) s, *p* < 0.0001; Arc Aβ + diazepam: 9.508 (8.363–10.20) s) and accuracy comparison (Kruskal–Wallis test with a Dunn’s multiple comparisons *post hoc* test; WT: 83.33 (66.66–100) % vs Arc Aβ + vehicle: 50 (33.33–50) %, *p* = 0.0008; Arc Aβ + vehicle vs Arc Aβ + XBD: 75 (50–87.5) %, *p* = 0.0102; Arc Aβ + diazepam: 66.66 (41.67–83.33) %) between the different treatment groups in the Retest 2 phase. WT (*n* = 9), Arc Aβ + vehicle (*n* = 9), Arc Aβ + XBD (*n* = 10) and Arc Aβ + diazepam (*n* = 9). **F** The escape latency and accuracy curves during the course of the 5-day training phase in the water cross maze for hetTSPOKO X Arc Aβ + Veh (n = 5) and hetTSPOKO X Arc Aβ + XBD (*n* = 5) groups. **G** Escape latency (Mann–Whitney *U* test: hetTSPOKO X Arc Aβ + XBD: 11.16 (8.168–14.46) s vs Arc Aβ + XBD: 7.420 (7.109–7.816) s, *p* = 0.0553) and accuracy comparison (Mann–Whitney *U* test: hetTSPOKO X Arc Aβ + XBD: 50.00 (37.50–54.17) % vs Arc Aβ + XBD: 66.66 (50.00–83.33) %, *p* = 0.0296) between the different treatment groups in the training phase. Arc Aβ + XBD (*n* = 10), and hetTSPOKO X Arc Aβ + XBD (*n* = 5) groups. **H** Escape latency (Mann–Whitney *U* test: hetTSPOKO X Arc Aβ + XBD: 9.800 (7.138–10.50) s vs Arc Aβ + XBD: 5.430 (4.844–6.237) s, *p* = 0.0047) and accuracy comparison (Mann–Whitney U test: hetTSPOKO X Arc Aβ + XBD: 50.00 (33.33–50) % vs Arc Aβ + XBD: 66.66 (50–66.66) %, *p* = 0.0033) between the different treatment groups in the Retest 1 phase. **I** Escape latency (Mann–Whitney *U* test: hetTSPOKO X Arc Aβ + XBD: 10.86 (8.2–14.24) s vs Arc Aβ + XBD: 5.608 (4.444–6.166) s, *p* = 0.008) and accuracy comparison (Mann–Whitney *U* test: hetTSPOKO X Arc Aβ + XBD: 50.00 (33.33–58.33) % vs Arc Aβ + XBD: 75 (50–87.5) %, *p* = 0.07) between the different treatment groups in the Retest 2 phase. Data are represented as median with their respective interquartile range. Data from Arc Aβ + XBD animals from the training phase, Retest 1 and Retest 2 (**C**–**E**) are used for comparison with hetTSPOKO X Arc Aβ (in G, H, I) *p < 0.05. ns: not significant.

Given the neuroprotective effects of XBD173 ex vivo, we asked whether XBD173 treatment in transgenic AD-modeled mice may improve cognitive deficits. The WCM was developed as a highly sensitive tool to assess hippocampal-dependent place learning in small animals [33]. Water as the only motive force and selective reinforcement paradigm results in more accurate and robust performances than food rewards [33]. These properties make this test highly suitable for detecting cognitive deficits developed in ArcAβ mice. The ArcAβ mice model of AD begins to develop robust cognitive impairment at an age group of 6–7 months [45]. In this study, we used two different treatment strategies to see whether XBD173 treatment can rescue these cognitive deficits. The treatment schedule of chronic and acute treated animals for the behavioral tests is shown in Fig. 3A and Supplementary Fig. 7A, respectively. The acute treatment schedule was started 2 days before the start of the training phase in the water cross maze while the chronic treatment of XBD173 (1 mg/kg) was followed for 12 weeks from the age of 8 months. In the acute treatment group mice treated with XBD173 had a comparable escape latency as well as accuracy to the vehicle-treated group (Supplementary Fig. 7A, B). Retests 1 and 2 which were accessed a week later and a month later respectively did not show any differences between the XBD173 treatment and the vehicle-treated group (Supplementary Fig. 7C, D). The training phase trajectories for both the escape latency and accuracy were comparable between the experimental groups (Supplementary Fig. 7A). This suggests that acute treatment of XBD173 might not be sufficient to improve spatial learning in the AD model of mice.

We followed up with a chronic treatment schedule to study whether chronic treatment of XBD173 could reverse the spatial learning deficits in the transgenic ArcAβ mice (Fig. 3A). Learning curves and final cognitive performance were markedly poorer in ArcAβ animals than in respective WT as assessed by escape latency and accuracy (Fig. 3B). Learning and accuracy curve trajectory in the training phase shows that chronic treatment of transgenic ArcAβ animals with XBD173 improves spatial learning in AD mice (Fig. 3B). Chronic XBD treatment significantly improves the escape latency right away in the training phase (Fig. 3C). While there was a tendency for higher accuracy in the XBD173-treated animals in the training phase, these differences were not significant compared to the vehicle-treated transgenic AD mice (Fig. 3C). Importantly, however, it is essential to note that the cognitive deficits were markedly rescued in both Retest 1 and Retest 2, performed after a week and a month respectively (Fig. 3D, E). The XBD173-treated ArcAβ in both the retests showed significantly better escape latency and accuracy compared to their vehicle-treated transgenic counterparts (Fig. 3D, E). This suggests a long-term neuroprotective effect of XBD173 on cognition even after terminating the treatment for more than a month. Since benzodiazepines are potent anxiolytics and are widely used in clinical practice, we included diazepam as a reference agent and applied this drug in an identical chronical regimen as XBD173. Unlike XBD173, diazepam did not show betterment in the spatial learning paradigm as monitored in the ArcAβ mice group (Fig. 3D, E). The escape latency and accuracy of the diazepam-treated transgenic AD mice were comparable to the vehicle-treated group. It is essential to note that changes in performance observed over the period of both retests were consistent among all animals. Specifically, any decline or improvement in comparison to the training phase was similar across the entire group (Supplementary Fig. 8A, B). Over the training phase and in both the retests, the XBD173-treated group showed a higher percentage of accurate learners compared to the vehicle-treated and diazepam-treated transgenic counterparts (Supplementary Fig. 8C–E).

To confirm the involvement of the TSPO protein in the XBD173-mediated neuroprotection in cognition, we crossbred the ArcAβ line with TSPOKO animals. The homozygous TSPOKO X ArcAβ line, however, showed behavioral saliences in the form of epileptic seizures. Therefore, we used the het TSPOKO X ArcAβ to investigate the role of the TSPO protein in conferring cognitive benefits. The het TSPOKO X ArcAβ animals treated with XBD173 did not show any marked improvements in cognitive performance compared to the vehicle-treated group as can be seen from both escape latency and accuracy parameters in the training phase as well as in both the retests (Supplementary Figs. 9, 10). The performance of XBD173-treated ArcAβ animals was significantly better than that of het TSPOKO X ArcAβ animals treated either with XBD173 or vehicle (Fig. 3F–I). The latency curve as well as the accuracy trajectory of hetTSPOKO X ArcAβ animals were poor as compared to the WT animals (Fig. 3F). These results indicate the neuroprotective effect of XBD173 on improving spatial learning in the AD mice model is dependent on the TSPO protein.

### Chronic XBD173 administration reduces plaque load, rescues the loss of dendritic spines, and increases neurosteroid levels in AD mice

**Fig. 4:**
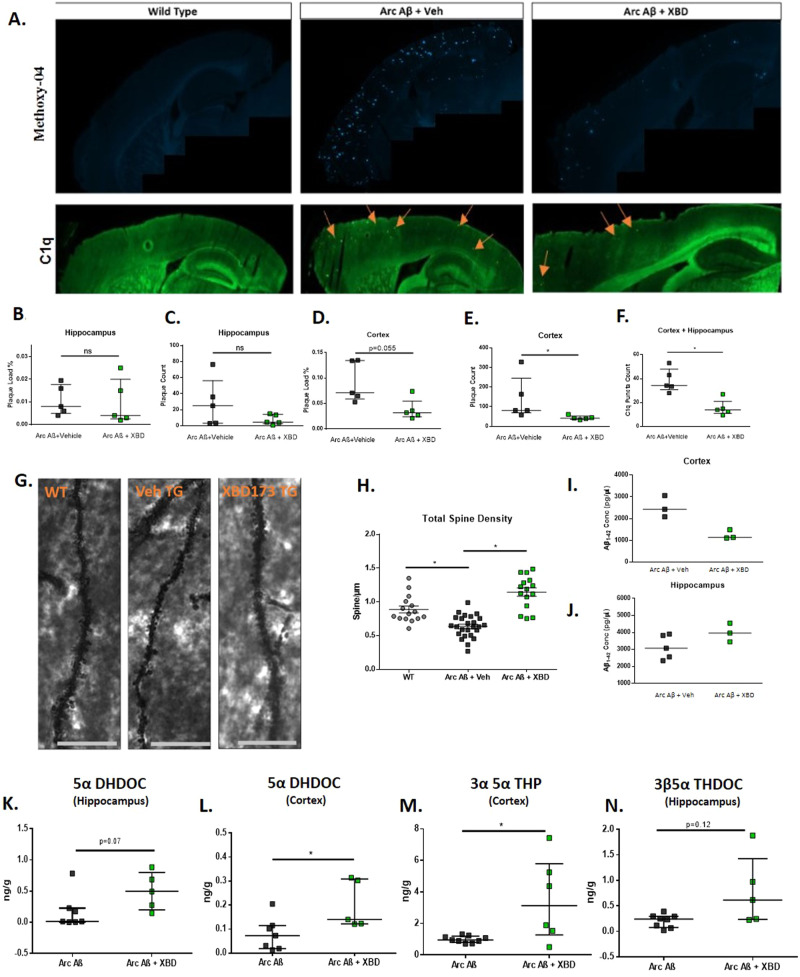
Chronic XBD173 administration reduces plaque load, rescues the loss of dendritic spines, and increases neurosteroid levels in AD mice. **A** Representative images for methoxy-04 plaque staining (first row) and C1q staining (second row) in WT, Arc Aβ + vehicle, Arc Aβ + XBD groups. **B** Plaque load % comparison between Arc Aβ + vehicle (*n* = 5) and Arc Aβ + XBD (*n* = 5) in the hippocampus (Mann–Whitney *U* test: Arc Aβ + vehicle: 0.0080 (0.0050–0.01775) % vs Arc Aβ + XBD: 0.0040 (0.0025–0.0200) %, *p* = 0.4524). **C** Plaque count comparison between Arc Aβ + vehicle (*n* = 5) and Arc Aβ + XBD (*n* = 5) in the hippocampus (Mann–Whitney *U* test: Arc Aβ + vehicle: 25 (3.25–56.25) vs Arc Aβ + XBD: 4.5 (2.5–14.30), *p* = 0.4127). **D** Plaque load % comparison between Arc Aβ + vehicle (*n* = 5) and Arc Aβ + XBD (*n* = 5) in the cortex (Mann–Whitney *U* test: Arc Aβ + vehicle: 0.0710 (0.0590–0.1345) % vs Arc Aβ + XBD: 0.0320 (0.0240–0.0550) %, *p* = 0.055). E. Plaque count comparison between Arc Aβ + vehicle (*n* = 5) and Arc Aβ + XBD (*n* = 5) in the cortex (Mann–Whitney *U* test: Arc Aβ + vehicle: 81.00 (69.50–246.3) vs Arc Aβ + XBD: 41 (36.15–52.50), *p* = 0.0159). **F** C1q aggregate count comparison between Arc Aβ + vehicle (*n* = 5) and Arc Aβ + XBD (*n* = 5) in the cortex and hippocampus (Mann–Whitney *U* test: Arc Aβ + vehicle: 34.33 (30.75–48.00) vs Arc Aβ + XBD: 14 (11–21), *p* = 0.0079). **G** Representative Golgi-Cox stained apical dendrites in the hippocampus for WT, Arc Aβ + vehicle, and Arc Aβ + XBD groups, respectively. **H** Total spine density calculated from *n* = 15–25 dendrites obtained from three to four mice per group (one-way ANOVA with a Bonferroni’s multiple comparisons *post hoc* test; WT: 0.8244 (0.7545–1.008) spines/µm vs Arc Aβ + vehicle: 0.6329 (0.5177–0.7629) spines/µm, *p* = 0.0005; Arc Aβ + vehicle vs Arc Aβ + XBD: 1.190 (0.9424–1.320) spines/µm, *p* < 0.0001). **I** Soluble Aβ_1-42_ levels were measured in the cortex. Arc Aβ + vehicle (*n* = 3), and Arc Aβ + XBD (*n* = 3). **J** Soluble Aβ_1-42_ levels measured in the hippocampus Arc Aβ + vehicle (*n* = 5), and Arc Aβ + XBD (*n* = 3). **K** 5α-DHDOC levels in the hippocampus for the treatment groups from GC-MS/MS analysis Arc Aβ + vehicle (*n* = 7) and Arc Aβ + XBD (*n* = 5) (Mann–Whitney *U* test: Arc Aβ + vehicle: 0.0150 (0.0060–0.2270) ng/g vs Arc Aβ + XBD: 0.4940 (0.2115–0.7860) ng/g, *p* = 0.07). **L** 5α-DHDOC levels in the cortex for the treatment groups from GC-MS/MS analysis Arc Aβ + vehicle (*n* = 7) and Arc Aβ + XBD (*n* = 5) (Mann–Whitney *U* test: Arc Aβ + vehicle: 0.0730 (0.0190–0.1150) ng/g vs Arc Aβ + XBD: 0.1400 (0.1220–0.3085) ng/g, *p* = 0.0177). **M** 3α 5α-THP levels in the cortex for the treatment groups from GC-MS/MS analysis Arc Aβ + vehicle (n = 9) and Arc Aβ + XBD (*n* = 6) (Mann–Whitney U test: Arc Aβ + vehicle: 0.9490 (0.8125–1.188) ng/g vs Arc Aβ + XBD: 3.127 (1.273–5.791) ng/g, *p* = 0.036). **N** 3β5*α*-THDOC levels in the hippocampus for the treatment groups Arc Aβ + vehicle (*n* = 8) and Arc Aβ + XBD (*n* = 5) (Mann–Whitney *U* test: Arc Aβ + vehicle: 0.2415 (0.076–0.2955) ng/g vs Arc Aβ + XBD: 0.6130 (0.2350–1.427) ng/g, *p* = 0.12). Data are represented as median with their respective interquartile range. *p < 0.05. ns: not significant.

To investigate the effects of chronic XBD173 treatment on AD pathology we looked at different parameters. We assessed the plaque load after staining with congo derivative methoxy-04 in the hippocampus and cortex of these mice. Interestingly, XBD173-treated mice showed a reduction of plaque load and count particularly in the cortex and not in the hippocampus (Fig. 4A–E). We also quantified soluble Aβ using an Aβ_1-42_-sensitive ELISA. The hemizygous overexpression of APP carrying the ArcAβ mutation increased Aβ_1-42_ levels. Consistent with the plaque load analysis, chronically applied XBD173 changed the amount of soluble Aβ_1-42_ as detected by ELISA only in the cortex (Fig. 4I, J). To understand whether chronic XBD173 treatment rescues the loss of dendritic spines, we analyzed the spine density by Golgi-cox staining. XBD173 treatment significantly rescued the loss of spines in transgenic mice (Fig. 4G, H). Further, GC-MS/MS analysis of XBD173-treated brains revealed an increase in several neurosteroid levels such as allopregnanolone (3α,5α-THP), 3β,5α-THDOC and 5α-DHDOC in the cortex and hippocampus (Fig. 4K–N). Together, these experiments demonstrate the disease-modifying effect of XBD173 in the AD mouse model. This hypothesis is further supported by reduced plaque load and soluble Aβ_1-42_ levels, rescued synaptic density, and increased neurosteroid levels.

### Chronic XBD173 treatment does not affect the resting or active microglial population markers

Previous studies have demonstrated a shift in microglial responsiveness from anti- to pro-inflammatory surface markers in AD [46, 47]. Microglia are a highly dynamic entity and can possess both pro- and anti-inflammatory markers [48]. However, in AD, the microglia have been shown to have an increased expression of inflammatory markers which include CD36, CD14, CD11c, MHCII, and iNOS [49–51]. Our study shows that transgenic ArcAβ mice exhibited a trend toward a reduction in the resting microglial population (Supplementary Fig. 11). Importantly, chronic XBD173 treatment did not appear to have any impact on the resting microglial population (Supplementary Fig. 11D). Additionally, we observed a tendency of increase in MHCII inflammatory markers in both vehicle and XBD treated ArcAβ mice in resting microglia (Supplementary Fig. 12), but no changes in inflammatory markers including CD80, IL-1β, and MHCII in activated microglia (Supplementary Fig. 13). This is similar to our ex vivo study where the addition of Aβ_1-42_ oligomers in hippocampal slices led to an increase in CD80 and CD163 expressing microglia. However, XBD173 treatment did not alter the increased CD80 or CD163 expressing microglial levels (Supplementary Fig. 14). Additionally, while there is an increased expression of microglial marker P2RY12 after the Aβ_1-42_ oligomers incubation, XBD173 doesn’t affect the increased levels of P2RY12 (Supplementary Fig. 14C). These findings suggest that XBD173 treatment does not affect microglial polarization states or alter the changes in the resting microglial population in ArcAβ mice.

### Chronic XBD173 treatment reduces the increased astrocytic-induced phagocytosis and pruning of synapses

**Fig. 5:**
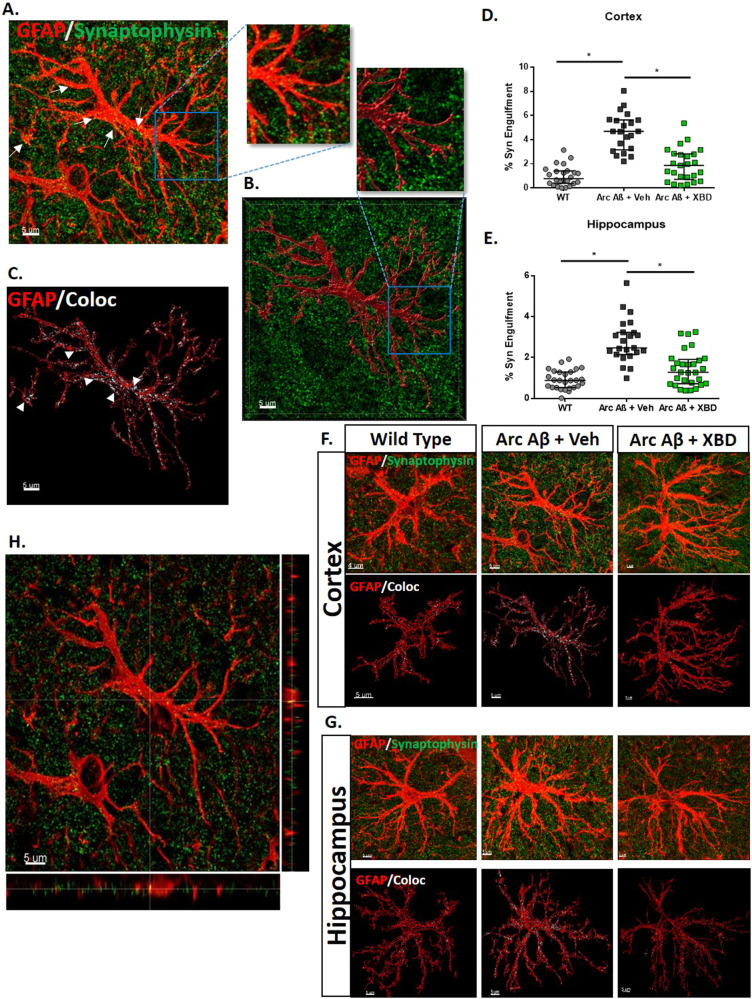
Chronic XBD173 treatment reduces astrocytic phagocytosis and pruning of synapses. **A** Individual high-resolution astrocytes (shown in red) and pre-synaptic marker synaptophysin (shown in green). The colocalization points are indicated by an arrow. **B** Volumetric reconstruction of astrocytes (red) using IMARIS 9.7 with synaptophysin (green). The insets show enlarged sections of before and after reconstruction of the astrocytes. **C** Rendered outline of astrocyte (red) with colocalization between astrocytes and synaptophysin marked in white and shown by the arrow. **D** % of Synaptophysin engulfment by astrocyte quantified in different treatment groups in the cortex (one-way ANOVA with a Bonferroni’s multiple comparisons *post hoc* test; WT: 0.7809 (0.3763–1.411) % vs Arc Aβ + vehicle: 4.692 (3.157–5.640) %, *p* < 0.0001; Arc Aβ + vehicle vs Arc Aβ + XBD: 1.866 (0.7195–2.818) spines/µm, *p* < 0.0001). **E** % of Synaptophysin engulfment by astrocyte quantified in different treatment groups in the hippocampus (Kruskal–Wallis test with a Dunn’s multiple comparisons *post hoc* test; WT: 0.8785 (0.5336–1.293) % vs Arc Aβ + vehicle: 2.468 (2.144–3.232) %, *p* < 0.0001; Arc Aβ + vehicle vs Arc Aβ + XBD: 1.276 (0.7177–1.906) %, *p* < 0.0001; 21–29 astrocytes collected from 4–5 mice per group). **F** Representative images for GFAP (red) and Synaptophysin (green) in the cortex in different experimental groups. The first panel shows the original image and the second panel shows the colocalization point (white) inside the rendered volume of the astrocyte from the respective groups. **G** Representative images for GFAP (red) and Synaptophysin (green) in the hippocampus in different experimental groups. The first panel shows the original image and the second panel shows the colocalization point (white) inside the rendered volume of the astrocyte from the respective groups. **H** Orthogonal plane showing the GFAP (red) and synaptophysin (green). Scale bar: 5 µm. Data are represented as median with their respective interquartile range. *p < 0.05. ns: not significant.

Astrocytes are known to actively eliminate synapses via synaptic pruning in developing and maturing neurons. This pruning process, however, is exacerbated in the case of AD pathophysiology. As such, we hypothesized whether chronic XBD173 treatment could reduce the increased astrocytic pruning in transgenic AD mice. When analyzing high-resolution individual astrocytes, we observed that chronic XBD173 treatment significantly reduced the enhanced synaptic engulfment by astrocytes in both the cortex and hippocampus (Fig. 5). Lower astrocytic phagocytosis could therefore be one of the potential factors responsible for a lower removal of functional synapses contributing to better cognitive function in XBD173-treated mice.

### Chronic XBD173 treatment decreases the C1q aggregates in AD mice and reduces the increase in astrocytic engulfment of C1q complement protein

**Fig. 6:**
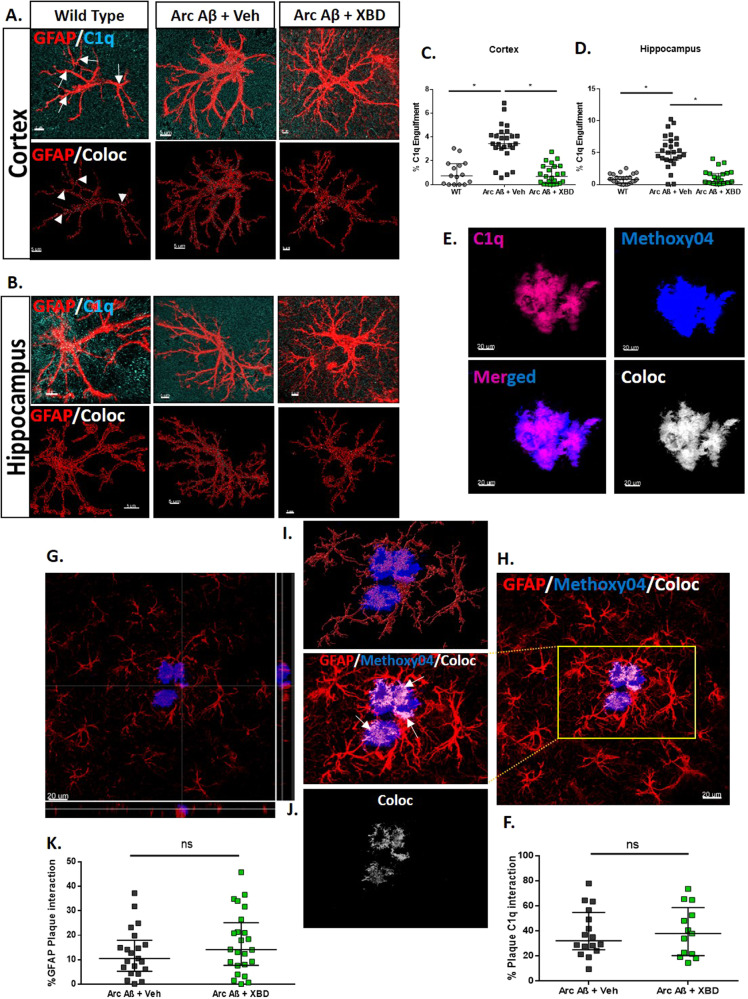
Chronic XBD173 treatment reduces the increase in astrocytic engulfment of C1q complement protein. **A** Representative images for GFAP (red) and C1q (cyan) in the cortex in different experimental groups. The first panel shows the original image and the second panel shows the colocalization point (white) inside the rendered volume of the astrocyte from the respective groups. Scale bar: 5 µm. **B** Representative images for GFAP (red) and C1q (Cyan) in the hippocampus in different experimental groups. The first panel shows the original image and the second panel shows the colocalization point (white) inside the rendered volume of the astrocyte from the respective groups. Scale bar: 5 µm. **C** % of C1q engulfment by astrocyte quantified in different treatment groups in the cortex (Kruskal–Wallis test with a Dunn’s multiple comparisons *post hoc* test; WT: 0.7465 (0.0033–1.767) % vs Arc Aβ + vehicle: 3.448 (3.071–4.300) %, *p* < 0.0001; Arc Aβ + vehicle vs Arc Aβ + XBD: 0.6829 (0.1004–1.543) %, *p* < 0.0001; 15–23 astrocytes collected from 4–5 mice per group). **D** % of C1q engulfment by astrocyte quantified in different treatment groups in the hippocampus (Kruskal–Wallis test with a Dunn’s multiple comparisons *post hoc* test; WT: 0.8102 (0.1865–1.250) % vs Arc Aβ + vehicle: 5.056 (3.849–6.986) %, *p* < 0.0001; Arc Aβ + vehicle vs Arc Aβ + XBD: 0.4372 (0.1979–1.733) %, *p* < 0.0001; 26 astrocytes per collected from 5 mice per group). **E** Representative 63X images of C1q aggregate (magenta), methoxy-04 plaques (blue), merged, and colocalization (white) in transgenic AD mice. Scale bar: 20 um. **F** % plaque C1q interaction in different treatment groups. (Mann–Whitney U test: Arc Aβ + vehicle: 32.18 (25.07–54.76) % vs Arc Aβ + XBD: 37.99 (20.39–58.68) %, *p* = 0.9390; 13–16 astrocytes collected from 4 mice per group). **G** Orthogonal plane representation of GFAP (red) and Plaque (blue) interaction **H**. Representative image for GFAP (red) and Plaque (blue) interaction. The inset shows an enlarged section of the interaction with colocalization marked by white arrows. **I** Reconstructed astrocytes (red) after removal of background signal along with methoxy-04 (blue). **J** The colocalization of GFAP and methoxy-04 plaque is shown in white. *Scale bar: 20* µm. **K** % plaque GFAP interaction in different treatment groups in the cortex and hippocampus. Arc Aβ + vehicle and Arc Aβ + XBD (Mann–Whitney *U* test: Arc Aβ + vehicle: 10.50 (5.305–17.99) % vs Arc Aβ + XBD: 14.15 (7.715–25.15) %, *p* = 0.3573; analysis from *n* = 21–24 plaques obtained from 4 mice per group) respective groups. **H** Orthogonal plane showing the GFAP (red) and synaptophysin (green). *Scale bar: 5* µm. Data are represented as median with their respective interquartile range. *p < 0.05. ns: not significant.

In physiological conditions, several studies have reported a C1q-independent astrocytic pruning of synapses [52]. However, in pathological conditions such as AD, there is strong evidence for C1q-dependent removal of synapses by the astrocytes [52]. To further understand the effects of XBD173 on astrocytic pruning in AD, we studied the connection of C1q with the astrocytic engulfment of synapses. We found deposition of C1q in the form of an aggregate that highly colocalized with amyloid plaques in the transgenic ArcAβ mice (Fig. 6E). However, while all the C1q aggregates were associated with the amyloid plaques, not all the plaques were associated with these aggregates. We asked whether chronic XBD173 treatment affects the colocalization percentage of C1q aggregates with the plaques. Colocalization of C1q aggregates with the plaques was comparable between both XBD173 and vehicle-treated groups (Fig. 6F). However, XBD173-treated group showed a significantly lower number of C1q aggregates as compared to their vehicle-treated counterparts (Fig. 4F).

C1q has been implicated as an “eat-me tag” for the neurons to undergo elimination. Since XBD173 treatment reduced the increased elimination of synapses by astrocytes, we hypothesized that individual astrocytes in the transgenic mice would subsequently engulf more of this C1q “eat-me tag” signatures and XBD173 treatment could affect this astrocytic engulfment of C1q. Consistent with this hypothesis, we observed increased engulfment of C1q by astrocytes in the vehicle-treated transgenic group. XBD173 treatment significantly reduced the astrocytic engulfment of C1q protein in the cortex and hippocampus (Fig. 6A–D; Supplementary Fig. 15). Additionally, our ex vivo experiment with slices also shows an enhanced astrocytic engulfment of C1q in the Aβ_1-42_ treated slice and XBD173 preincubation prevents this (Supplementary Fig. 16). Taken together, these results highlight C1q aggregate as an additional biomarker for AD and show that XBD173 treatment could rescue the exacerbated C1q capture by astrocytes.

### Astrocytic phagocytosis does not contribute to the clearance of methoxy plaques in the cortex

In view of the decreased plaque load in the cortex of XBD173-treated animals and its effect on astrocytic phagocytosis, we asked whether astrocytic phagocytosis of plaques may result in reduced plaque load in these animals. We investigated the number of plaques inside the surrounding astrocytic volume. Astrocytic density around the plaques (at a radius of 30 µm from the plaques) was higher than more away from the plaques. We found that the astrocytic engulfment of plaque was comparable for both vehicle and XBD173-treated animals (Fig. 6G–K). These results indicate that astrocytic phagocytosis does not directly contribute to the removal/reduced plaque load in XBD173-treated transgenic AD mice.

## Discussion

The TSPO ligand XBD173 (AC-5216/emapunil) has been previously shown to exert rapid anxiolytic effects in animal models and humans [28]. In this study, we provide both in vitro and in vivo evidence for the neuroprotective effect of XBD173 in AD via the TSPO protein. In our study, we show that XBD173 confers neuroprotection against Aβ_1-42_ oligomers and partly rescues the LTP impairment occurring from Aβ_1-40_ oligomers. This differential action of XBD173 could be attributed to the mechanistic differences in how both Aβ_1-42_ and Aβ_1-40_ act to inhibit the formation of CA1 LTP in the hippocampus. A previous study with soluble Aβ oligomers shows the overactivation of NR2B subunit-containing NMDA receptors as the primary contributor to LTP impairment [53]. Taken together, it is plausible to hypothesize that XBD173 incubation prevents the NMDA excitotoxicity resulting from Aβ oligomers. In addition, XBD173 rescued the loss of mushroom spines as well as the long thin spines. While mushroom spines because of a higher head volume are thought to play a critical role in long-term memory-related functions, long thin spines because of their adaptability are also called learning spines [54]. This suggests that the synaptic dysfunction of neurotransmission resulting from the Aβ_1-42_ oligomer is prevented by the administration of XBD173.

TSPO plays an important role in transferring cholesterol from the outer to the inner membrane and plays an important role in neurosteroidogenesis. Recently, however, some studies have challenged its role in the synthesis of neurosteroids. This is supported by the knockout models where TSPO knockout does not affect the phenotype or viability of the animal line [30]. This argument, however, is countered by the upregulation of steroid levels by TSPO ligands as well as a study from Owen et al., which shows impaired steroid synthesis in the absence of TSPO [55]. Furthermore, in vitro expression and site-directed mutagenesis of the TSPO protein reveal a cholesterol-recognizing amino acid consensus domain (CRAC) in its cytosolic C terminus [16]. Additionally, TSPO deficiency resulted in an impairment of local neurosteroidogenesis in the brain. Consistent with the role of TSPO in neurosteroidogenesis, we observed elevated levels of 3α5α-THP in the cortex, 3β5α-THDOC in the hippocampus, and 5α-DHDOC levels in the cortex and hippocampus of the chronic XBD-treated animals. This suggests that XBD173 might affect a multitude of neurosteroids via TSPO and these neurosteroids target a multitude of pathophysiological features of AD. We, therefore, studied the neuroprotective effect of neurosteroids in preventing Aβ_1-42_ mediated LTP deficit. Allopregnanolone prevents LTP impairment resulting from the Aβ_1-40_ oligomer. On the other hand, XBD173 was able to partly rescue the LTP impairment from Aβ_1-40_ oligomers. This could be explained by a shorter duration/concentration of XBD173 in an ex vivo setup which might not produce desirable protection as 100 nM allopregnanolone when applied directly to the bath solution. In other terms, it could be that 300 nM XBD173 does not produce sufficient allopregnanolone ex vivo necessary to exhibit positive action. Moreover, certain 3β neurosteroids are known for their functional antagonism of 3α-reduced neurosteroids at GABA_A_ receptors. [56]. We found that 3β,5α-THDOC, which was elevated after chronic treatment of XBD173, also prevents the LTP disruption resulting from Aβ_1-42_ oligomers. Additionally, its stereoisomer 3α,5α-THDOC (100 nM) similar to XBD173 prevented the LTP impairment from the Aβ_1-42_ oligomer. Previously, THDOC was found to inhibit acetylcholinesterase which is significantly elevated in AD [57].

The extrasynaptic benzodiazepine-insensitive GABA_A_ receptors containing the δ-subunits are considered important targets for neurosteroids. In our study, we found that knocking out the δ-subunit of the GABA_A_ receptor impairs the neuroprotective effect of 3α,5α-THDOC against the Aβ_1-42_ oligomer. Importantly, XBD173 similar to THDOC could not prevent the LTP impairment from Aβ oligomer in the absence of δ-subunit containing GABA_A_ receptors. These results provide two essential insights. 1. TSPO-dependent XBD173 acts via the GABA_A_ receptor containing the δ-subunit to exert its neuroprotective effects. 2. The downstream activity of XBD173 is possibly mediated by neurosteroids such as THDOC which acts via the δ-subunit. As such, Aβ has opposing effects on the NMDA receptors in contrast to APP i.e., Aβ increases the glutamate concentration in the synaptic cleft and has an agonistic effect on the NMDA receptors [58]. Contrary to the physiological state where the NMDA receptors need strong depolarization of the postsynaptic membrane by glutamate (mM conc.) for the removal of voltage-dependent Mg^2+^ ion blockade, in AD, NMDA receptors are activated for longer durations by a much lower concentration of glutamate (µM conc.) [58]. LTP or synaptic plasticity largely depends on the detection of relevant signals over the background noise from the moderate intracellular Ca^2+^ signal (higher signal-to-noise ratio). This signal-to-noise ratio however is impaired in the presence of the Aβ oligomers due to increased noise from the chronically overactive glutamatergic system and impaired Mg^2+^ filter in the NMDA receptors [58]. Previously, it has been reported that extrasynaptic GABA_A_ receptors are well suited to antagonize the over-activation of NMDA receptors on pyramidal cells [59]. Several characteristics make GABA_A, slow_ well suited to antagonize the over-activation of NMDA receptors on pyramidal cells [60]. First, their slow time-course matches that of NMDA receptor-mediated synaptic currents [61]. In addition, the location of axonal endings of neurogliaform cell interneurons (NGF-INs) mediating GABA_A, slow_ is closely associated with the major afferent glutamatergic inputs on the dendrites of hippocampal pyramidal neurons [60]. Furthermore, volume transmission by NGF-INs activates extrasynaptically located GABA_A_ receptors, which antagonize voltage-dependent opening of extrasynaptic NMDA receptors. Moreover, GABA_A, slow_ can suppress calcium-dependent spiking and sodium spike back-propagation from the soma to the dendrites. Multiple subtypes of GABA_A_ receptors contribute to the generation of GABA_A,slow_ most probably including extrasynaptic GABA_A_ receptors incorporating δ-subunits [61, 62]. Taken together, one could speculate that XBD173 modulation of the δ subunit GABA_A_ receptor could down tune this background noise from the overactivated glutamatergic system in the presence of Aβ oligomers. This would therefore also relieve the energy burden due to increased neuronal activity in the presence of Aβ oligomers. Additionally, the modulation of δ subunit GABA_A_ receptor activity by XBD173 could also impact the amyloidogenic processing of APP.

The water cross maze provides conclusive evidence that XBD173 betters spatial learning in the transgenic ArcAβ mice and is dependent on the TSPO to mediate this beneficial action. This, however, challenges the recent reports from Shi et al., which suggest benzodiazepines and TSPO ligands alter synaptic plasticity and cause cognitive impairment [26]. Respective differences could be due to the differences in experimental conditions used in both studies. First, the pathological expression of TSPO is different from the physiological state [63, 64]. As described in the discussion section earlier, XBD173 could down tune the noise from tonically overactive glutamatergic signal in AD via GABA_A_ containing delta subunit, thereby leading to a better plasticity/learning profile. In a physiological condition as studied by Shi et al., [26], however, this could lead to the imbalance of the glutamatergic and GABAergic systems, leading to impaired plasticity. This falls in line with our observation of control chronic treatment groups where the wild-type mice chronically treated with XBD173 have lower accuracy in Retest 2 (not significant) as compared to the untreated counterparts (Data not shown). Second, the dose of XBD173 (5 mg/kg) used by Shi et al., [26], as well as the duration (consecutive days), varies considerably from our study (1 mg/kg every alternate day). In line with these hypotheses, a recent study showed that XBD173 at a higher dosage (5 mg/kg) compared to the lower dosage (1 mg/kg) affects the mouse locomotory and exploratory behavior and also decreases the gamma power in EEG recordings [27]. Given the importance of gamma power in maintaining working memory-related processes, one could speculate that a decrease in gamma power resulting from a higher dosage of XBD173 impairs cognitive functions. Additionally, XBD173 has a long residence time at the TSPO binding site and a previous study with a mouse multiple sclerosis model suggests that repeated administration of a low dose of XBD173 is beneficial in clinical and pathological outcomes as compared to a higher dose [65, 66]. It is therefore essential to note that both hypo- and hyper-activation of the glutamatergic system leads to neuronal dysfunction and there has to be a subtle balance in any therapeutics that are designed for AD [58]. Our observation of epileptic seizures in homozygous TSPOKO X ArcAβ could possibly be attributed to several factors. First, the development of seizures could stem from the genetic interaction between TSPOKO and the ArcAβ line. Additionally greater mutational burden could influence the seizure development. Second, the differences in the type and extent of Aβ pathology between different Alzheimer’s models could contribute to variations in neurological outcomes. Third, from previous clinical studies Aβ derived pathology induces seizures and a deficiency of TSPO could increase the risk of developing seizures.

Chronic administration of XBD in the ArcAβ decreased the plaque load, particularly in the cortex, and also reduced the soluble Aβ levels in the cortex. This effect was not seen in the hippocampus. The transgenic ArcAβ mice did not form extensive plaques in the hippocampus region even at the age of around 12 months old. The TSPO expression pattern is not homogeneous and differs across the brain regions, adding to it the cellular expression of TSPO in a pathologic state, which could be the reason for regional differences. A decrease in plaque load and plaque count in the cortex of XBD-treated animals indicates either an increased clearance of the plaques or protection machinery that avoids plaque formation. Chronic XBD173 treatment also rescued spine loss and thereby preserved functional spines. The reduction of functional spine loss could be attributed either to a reduced amyloid pathology or to a reduction in astrocytic phagocytosis.

Loss of functional synapses due to injury or disease affects neuronal communication and leads to network disruption in the central nervous system (CNS). Reactive astrogliosis has often been characterized as a hallmark feature of AD [67]. Previously, TSPO has often been considered to be a microglial activation marker and is shown to be overexpressed in neuroinflammatory conditions. However, recent studies with TSPO have highlighted that increased TSPO expression in neurological disorders does not necessarily indicate upregulation of TSPO, instead, it’s associated with increased glial density resulting in higher TSPO signals [64, 68, 69]. This aligns with our study findings; we observe no alterations in TSPO expression within glial cells in ArcAβ mice (Supplementary Fig. 11A–C). Additionally, in contrast to previous ideas of TSPO levels being influenced only in inflammatory conditions or in glial cells, recent findings suggest that neuronal activity can influence the TSPO levels in the brain [70, 71].

Previous reports show impairment of the astrocytic phagocyte receptors Multiple EGF Like Domains 10 (MEGF10) and Mer Tyrosine Kinase (MERTK) in the murine AD model, which implies an inefficient clearance of senile plaques [72]. We, therefore, asked whether the reduction in the number of amyloid plaques in the cortex region of XBD173-treated mice was due to better phagocytic clearance of these amyloid plaques. Interestingly, we observed that TSPO activation does not significantly change the GFAP-stained astrocytes-plaque interaction, which suggests a similar astrocytic recruitment profiling around the plaque. Moreover, the uptake of the plaque by the astrocyte is not altered by the treatment with XBD173. This suggests that TSPO activation by XBD173 does not affect the phagocytic clearance of the senile plaque but instead confers neuroprotection by interfering with the plaque formation machinery and stopping the formation of senile plaques. A second possible way would be the activation of microglia either directly by XBD173 or indirectly via astrocytes to clear up the amyloid plaques.

The complement system together with glial cells is crucial for mediating early synapse loss thereby driving the pathophysiology of AD [73–75]. In the developing neurons, C1q and C3 of the complement pathway localize to synapses and facilitate the refining of synapses by pruning [76]. This process however becomes aberrant in AD pathology with several reports suggesting an abnormal increase in C1q levels of the murine model of AD [73]. MEGF10, which is expressed on the astrocytic surface, is a receptor for C1q, which in turn attaches to the phosphatidylserine expressed on the apoptotic cell surface [77]. Previously, C1q deletion has been associated with a marked reduction in astrocyte-synapse association, thereby rescuing the synaptic density [52]. Similarly, injection of oligomeric Aβ has been shown to increase the association of C1q and postsynaptic markers [73, 78]. C1q has also been reported to have a specific binding site for Aβ [79]. Both C1q and oligomeric Aβ act in an overlapping way to eliminate the synapses and treatment of the hippocampal slices with anti-C1q antibody significantly rescues the impairment in the LTP caused by the oligomeric Aβ [73, 80]. All these studies point towards C1q playing a crucial role as a destruction signal protein in synapse elimination. For these reasons, we studied whether the activation of TSPO protein by XBD173 and its effect on reduced astrocytic phagocytosis is related to the astrocytic engulfment of C1q “eat-me tags” in the AD model. In the current study, we found that the astrocyte engulfment of C1q is enhanced in the hippocampus and the cortex of the AD mouse model. This possibly explains the increase in the loss of functional synapses and an impairment of cognitive function that is evident from the behavioral tests. Chronic treatment of XBD173 decreased this increased engulfment of C1q “eat me tags” in the hippocampus. The reduction in astrocytic engulfment of both presynaptic marker (synaptophysin) and eat-me tag (C1q) suggests that the XBD173 treatment directly reduces the abnormal loss of functional synapses. Taken together, the results from our study as well as previous reports support the hypothesis that C1q is an interesting target for developing effective therapeutics in AD. Targeting of C1q not only affects the proinflammatory status of glial cells but also affects the cross-communication between astrocytes and microglia. Previously, C1q expression in microglia has been observed to increase in different models of AD, and TSPO ligands are shown to reduce this higher expression of microglial C1q [73, 81]. In contrast to these studies, we did not find any effect of TSPO ligand XBD173 on microglial C1q levels in ArcAβ mice model.

We also noted the deposition of C1q in the form of aggregate heavily in the cortex of the transgenic AD mice. These C1q aggregates highly overlap with the Aβ plaques [82]. While the report of colocalization of amyloid plaques with C1q of the complement pathway remains scarce, one of the previous studies shows distinct colocalization of Aβ protein with C3 complement protein in certain substructural domains within drusen in the retina [83]. Few of the previous reports have also shown a colocalization of both C4 binding protein as well as factor H of the complement pathway with the amyloid plaques and dead cells in the AD brain [84]. Therefore, the aggregate formation could be explained by the deposition of dystrophic neurites. It is interesting to note that not all the plaques overlap with these C1q aggregates but all the C1q aggregates do overlap with amyloid plaques. This might be of relevance because first, this might act as an additional diagnostic marker to amyloid plaque, and second, this could give us an overview/role of complement-mediated inflammation in the pathophysiology of AD. Taken together, the complement protein C1q may play play a critical role in pathogenesis of AD.

**Fig. 7:**
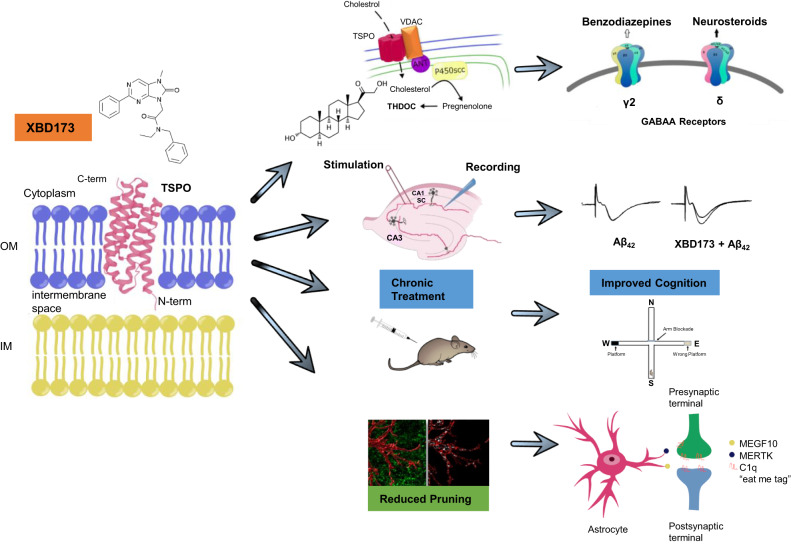
Proposed working mechanism for TSPO-dependent XBD173.

In conclusion, the present study suggests the neuroprotective properties of XBD173 in a mouse model of AD. Nevertheless, some limitations should be considered. First, while the ArcAβ mice model recapitulates the major phenotypes of AD including plaque formation, cognitive impairment, or reactive gliosis, it lacks the presence of neurofibrillary tangles which have also been shown to play an important role in cognitive decline in AD. Second, one could perceive the anxiolytic effect generated by XBD173 administration as a primary mediator of improvement in the cognitive deficit in the water cross maze. While it is difficult to completely separate the neuroprotective from the anxiolytic effects, the performance of XBD173-treated animals in two different retests tested months apart even after the termination of the treatment points towards the beneficial effects on cognitive performance. Moreover, it is essential to note that XBD173 improves the LTP deficit in the ex vivo hippocampal slices which is a cellular correlate of the learning and memory function. It is important to note that the procognitive benefits of XBD173 could be attributed to several factors which include XBD173-induced plaque reduction, soluble Aβ levels, and reduced synaptic pruning. In summary, the present results indicate the beneficial effects of XBD173 against Aβ-derived pathology (Fig. 7). Chronic XBD173 treatment ameliorates cognition and suggests a disease-modifying effect when applied at the early stages of AD.

### Supplementary information

Supplementary Information
