# Supplementary material for: Chronic administration of XBD173 ameliorates cognitive deficits and neuropathology via 18 kDa translocator protein (TSPO) in a mouse model of Alzheimer’s disease
Source: Transl Psychiatry. 2023 Oct 27;13:332. doi: 10.1038/s41398-023-02630-z (PMC10611770; doi:10.1038/s41398-023-02630-z)
Supplement: Supplementary file 1 — Supplementary Information [file 41398_2023_2630_MOESM1_ESM.pdf]

Supplementary Information

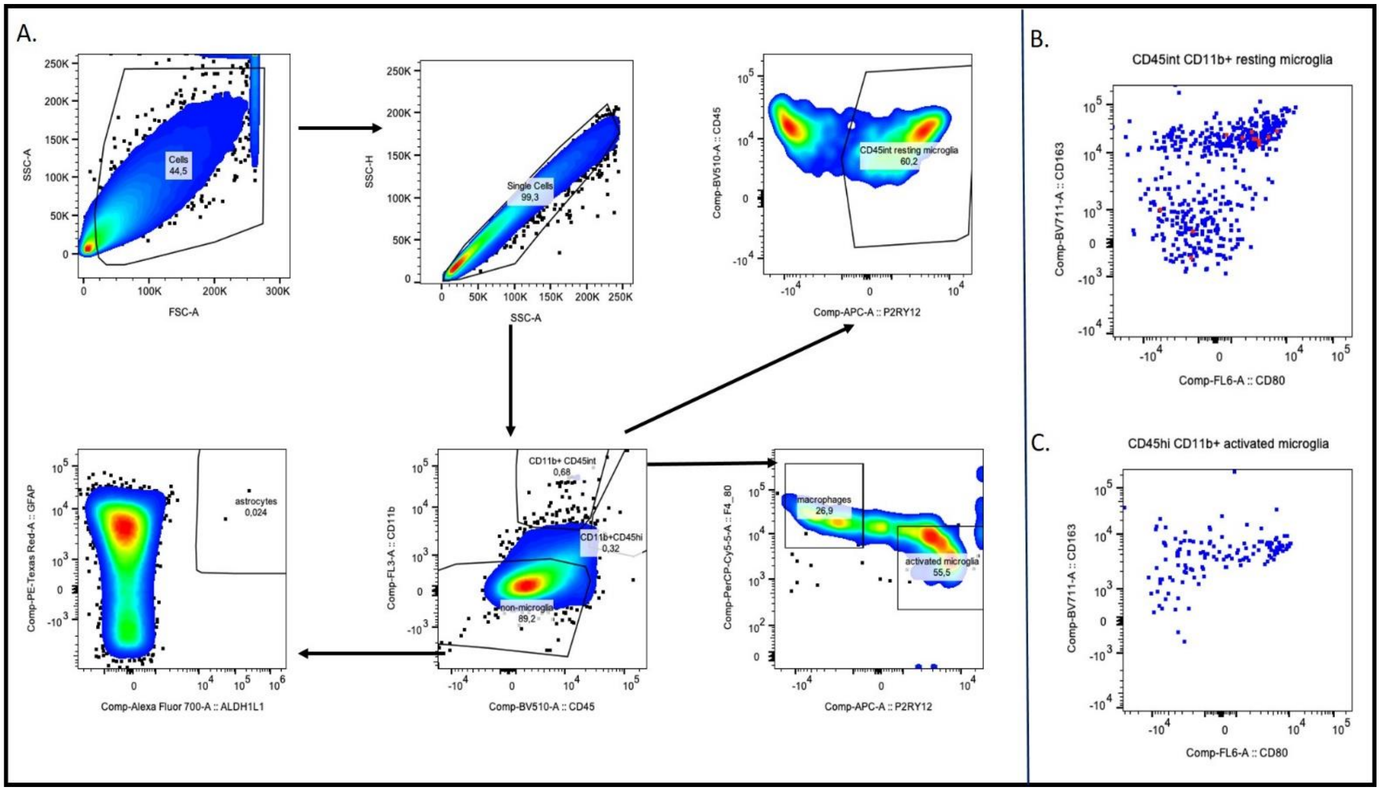

Supplementary Figure 1: A. Gating strategy to determine microglia and astrocyte population in chronically treated animals. B. Scatter dot plot of CD80 vs. CD163 for resting microglia. C. Scatter dot plot of CD80 vs. CD163 for activated microglia.

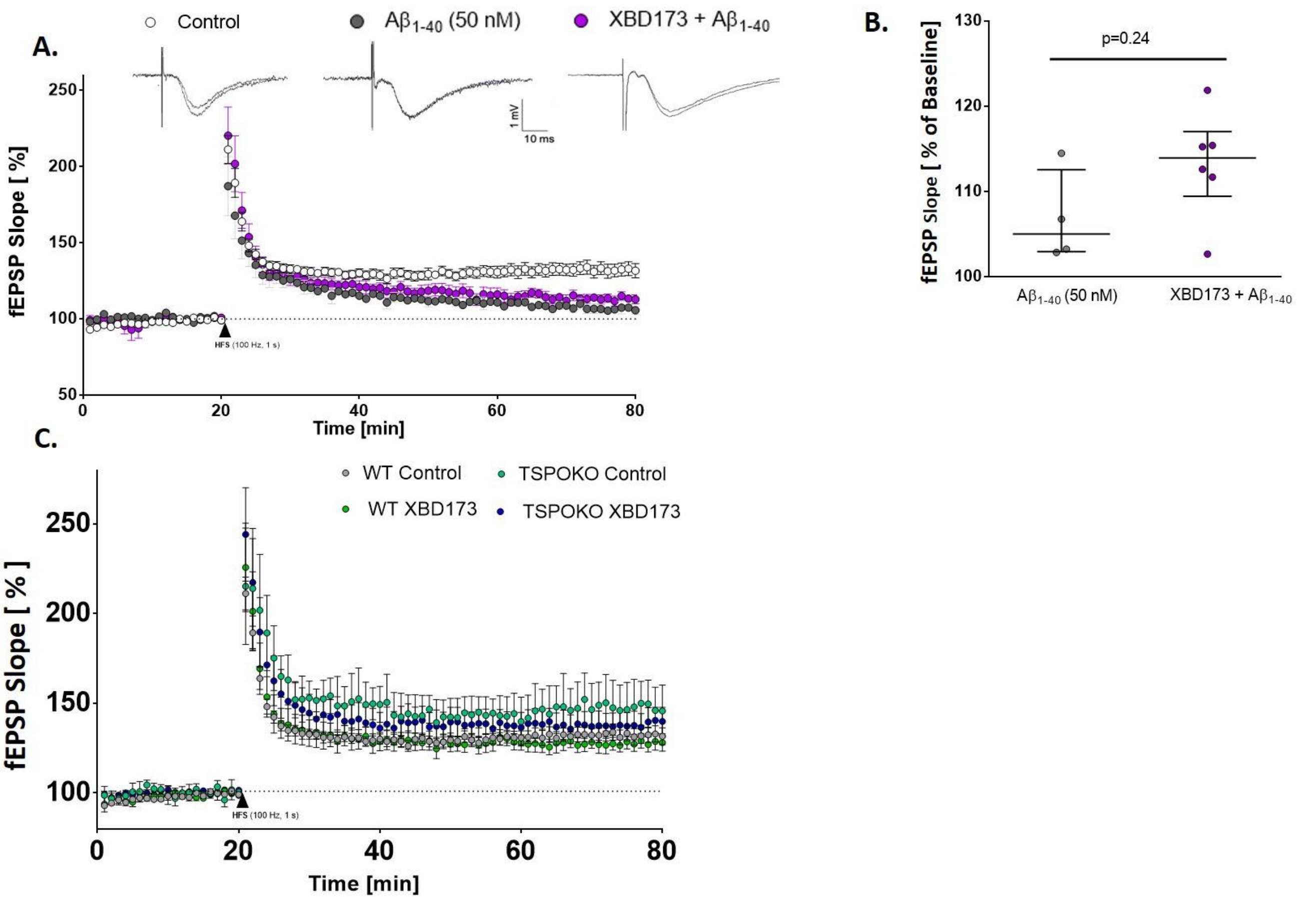

Supplementary Figure 2: XBD173 partly rescues the LTP impairments resulting from $A\beta_{1-40}$ oligomers. A. Normalized field excitatory postsynaptic potential (fEPSP) time course following a high-frequency stimulation (HFS) under control conditions, with 90 min $A\beta_{1-40}$ exposure alone, and the simultaneous application of XBD173 (300 nM) and $A\beta_{1-40}$ respectively. The inlets on the top are representative traces for each treatment group. Control (n=10/8 [n=slices from animals]), $A\beta_{1-40}$ (n=4/4) and XBD173 + $A\beta_{1-40}$ (n=6/6) B. Scatter dot plot summarizing the last 10 min (starting from 50 min to 60 min) after HFS for respective groups. Control (n=10/8 [n=slices from animals]), $A\beta_{1-40}$ (n=4/4) and XBD173 + $A\beta_{1-40}$ (n=6/6) (Mann-Whitney U test; $A\beta_{1-40}$: 105 (102.9-112.5) % of baseline slope vs XBD173 + $A\beta_{1-40}$: 113.9 (109.4-117) % of baseline slope, p=0.24). C. Normalized field excitatory postsynaptic potential (fEPSP) time course following a high-frequency stimulation (HFS) under WT Control (n=9/9), TSPOKO Control (n=5/5), WT XBD173 (n=6/6), TSPOKO XBD173 (n=6/6). Data are represented as median with their respective interquartile range. *$p < 0.05$. ns: not significant.

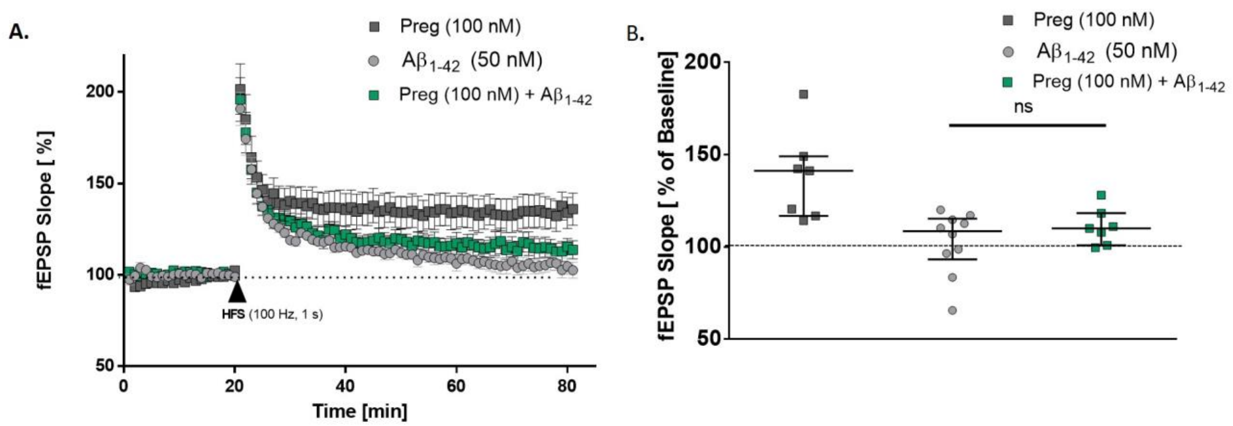

Supplementary Figure 3: Pregnenolone doesn't rescue the LTP impairments resulting from A$\beta_{1\text{-}42}$ oligomers. A. Normalized field excitatory postsynaptic potential (fEPSP) time course following a high-frequency stimulation (HFS) under Preg (100 nM) (n=7/6 [n= slices from animals]), A$\beta_{1\text{-}42}$ (50 nM) (n=10/10) and Preg+ A$\beta_{1\text{-}42}$ (n=7/6). B. Scatter dot plot summarizing the last 10 min (starting from 50 min to 60 min) after HFS for respective groups in WT C57/Bl6 mice: Preg (100 nM) (n=7/6 [n= slices from animals]), A$\beta_{1\text{-}42}$ (50 nM) (n=10/10) and Preg+ A$\beta_{1\text{-}42}$ (n=7/6) (Mann-Whitney U test; A$\beta_{1\text{-}42}$: 108.5 (93.18-115.3) % of baseline slope vs Preg (100 nM) + A$\beta_{1\text{-}42}$: 110 (100.8-118.3) % of baseline slope, p=0.47). Data are represented as median with their respective interquartile range. *$p < 0.05$. ns: not significant.

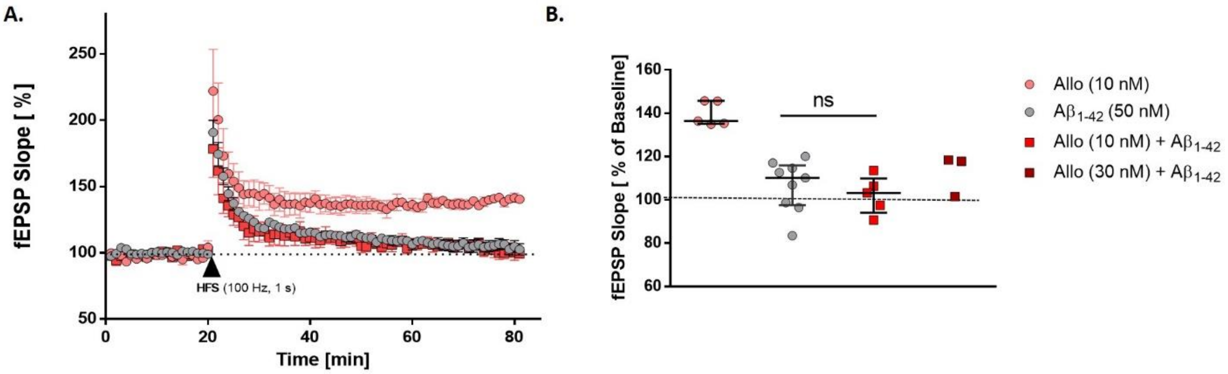

Supplementary Figure 4: Allopregnanolone doesn't rescue the LTP impairments resulting from Aβ$_{1-42}$ oligomers. A. Normalized field excitatory postsynaptic potential (fEPSP) time course following a high-frequency stimulation (HFS) under Allo (10 nM), Aβ$_{1-42}$ (50 nM), Allo (10 nM) + Aβ$_{1-42}$, and Allo (30 nM)+ Aβ$_{1-42}$. B. Scatter dot plot summarizing the last 10 min (starting from 50 min to 60 min) after HFS for respective groups in WT C57/Bl6 mice: Allo (10 nM) (n=5/5 [n=slices from animals]), Aβ$_{1-42}$ (50 nM) (n=9/9), Allo (10 nM)+ Aβ$_{1-42}$ (n=5/5), and Allo (30 nM)+ Aβ$_{1-42}$ (n=3/3) (Mann-Whitney U test; Aβ$_{1-42}$: 110.2 (97.55-115.9) % of baseline slope vs Allo (10 nM) + Aβ$_{1-42}$: 103.2 (94.08-109.9) % of baseline slope, p=0.34). Data are represented as median with their respective interquartile range. *$p < 0.05$. ns: not significant.

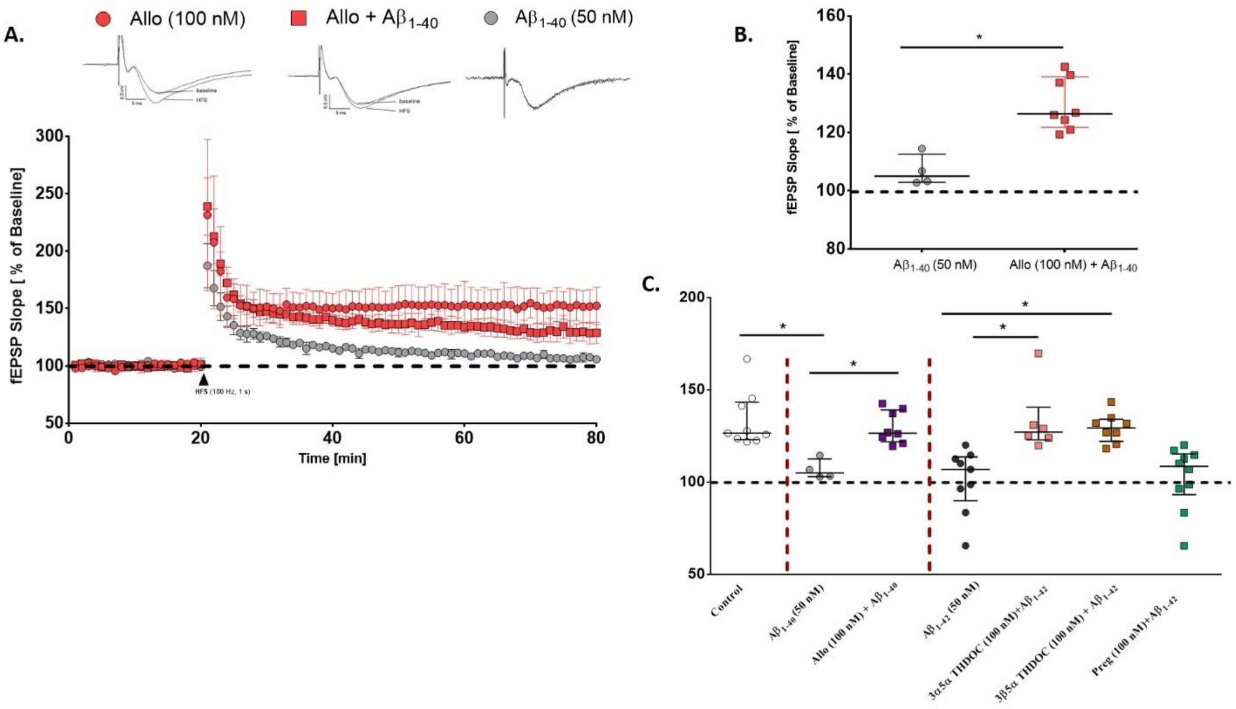

Supplementary Figure 5: Allopregnanolone rescues the LTP impairments resulting from $A\beta_{1-40}$ oligomers. A. Normalized field excitatory postsynaptic potential (fEPSP) time course following a high-frequency stimulation (HFS) under Allo (100 nM), $A\beta_{1-40}$ (50 nM) and Allo + $A\beta_{1-40}$. B. Scatter dot plot summarizing the last 10 min (starting from 50 min to 60 min) after HFS for respective groups in WT C57/Bl6 mice: $A\beta_{1-40}$ (50 nM) (n=4/4 [n=slices from animals]) and Allo (100 nM) + $A\beta_{1-40}$ (n=8/8) (Mann-Whitney U test; $A\beta_{1-40}$: 105 (102.9-112.5) % of baseline slope vs Allo (100 nM) + $A\beta_{1-40}$: 126.4 (121.8-139.1) % of baseline slope, p=0.004). C. Summary graph showing the effect of different neurosteroids on Aβ mediated CA1-LTP impairment. Data are represented as median with their respective interquartile range. *$p < 0.05$. ns: not significant.

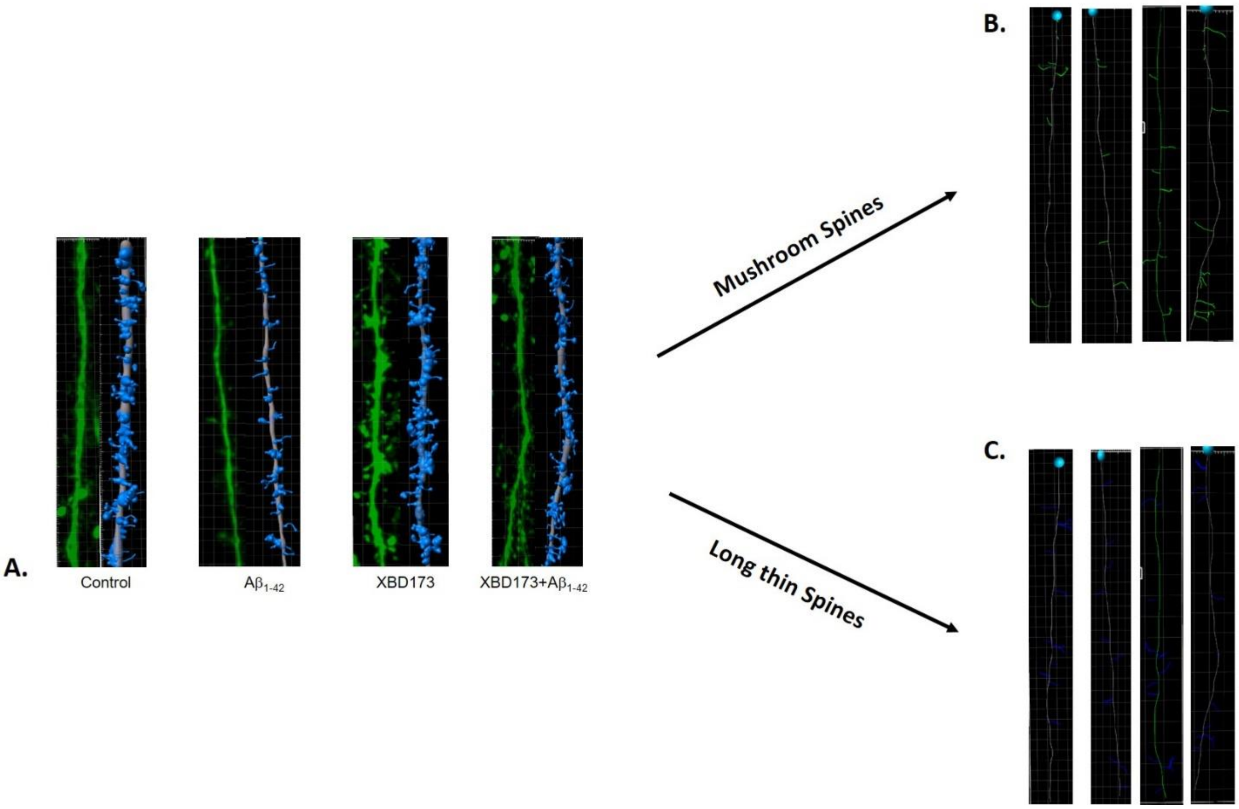

Supplementary Figure 6: A. Representative images of dendritic spines along with its rendered image from IMARIS. Control (n=6), $A\beta_{1-42}$ (n=5), XBD173 (n=4), and XBD173 + $A\beta_{1-42}$ (n=4) B. After categorization into Mushroom spines for different treatment groups. C. After categorization into long thin spines for different treatment groups.

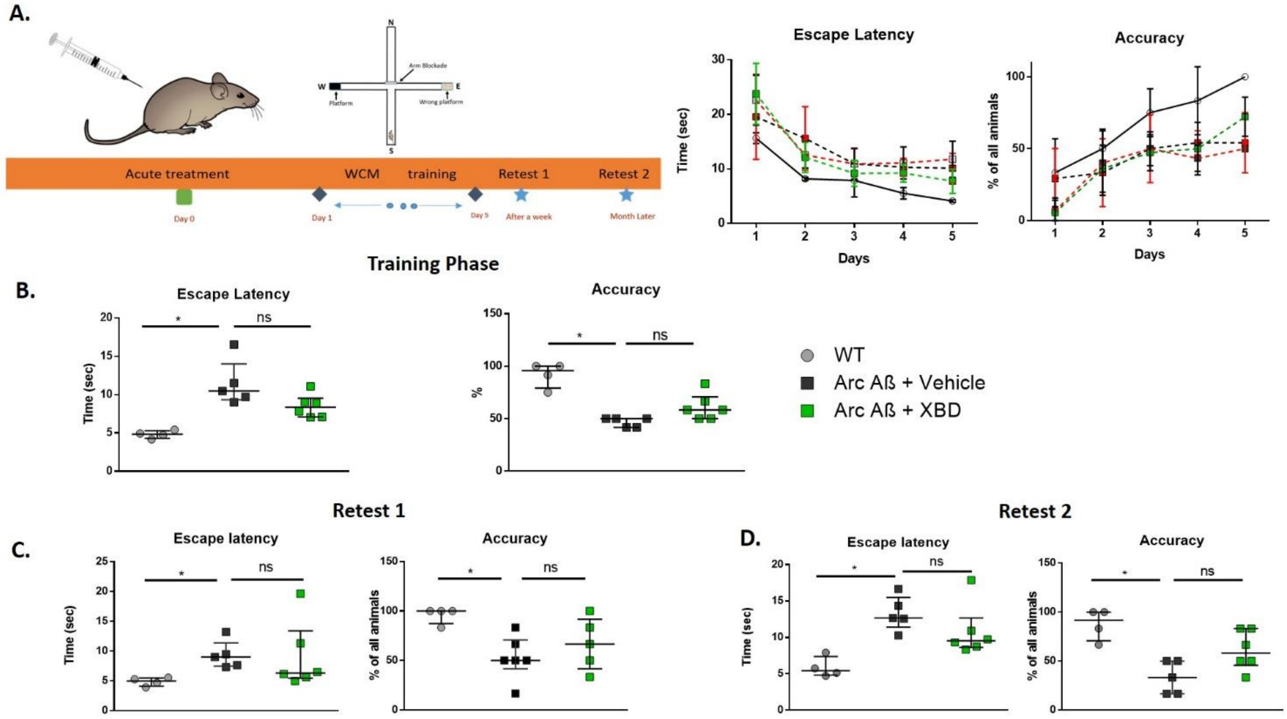

Supplementary Figure 7: Acute administration of XBD173 doesn't ameliorate cognitive deficits in AD mice. A. Schematic of acute treatment schedule, training, and retests in water cross maze. Adjacent to the schematic are the escape latency and accuracy curves during the 5-day training phase in the water cross maze. B. Escape latency (Kruskal–Wallis test with a Dunn's multiple comparisons *post hoc* test; Arc Aβ + Vehicle: 10.47 (9.345-14.02) s vs Arc Aβ + XBD: 8.355 (7.095-9.548) s, p=0.27) and accuracy comparison (Kruskal–Wallis test with a Dunn's multiple comparisons *post hoc* test; Arc Aβ + Vehicle: 50 (41.67-50) % vs Arc Aβ + XBD: 58.33 (50-70.84) %, p=0.17) between the different treatment groups in the training phase. WT (n=4), Arc Aβ + Vehicle (n=5), and Arc Aβ + XBD (n=6). Accuracy is expressed for each animal as the % of trials correctly performed, C. Escape latency (Kruskal–Wallis test with a Dunn's multiple comparisons *post hoc* test; Arc Aβ + Vehicle: 9.01 (7.473-11.36) s vs Arc Aβ + XBD: 6.302 (5.488-13.39) s, p=0.69) and accuracy comparison (Kruskal–Wallis test with a Dunn's multiple comparisons *post hoc* test; Arc Aβ + Vehicle: 50 (41.67-70.83) % vs Arc Aβ + XBD: 66.66 (41.67-91.67) %, p=0.82) between the different treatment groups in the Retest 1 phase. D. Escape latency (Kruskal–Wallis test with a Dunn's multiple comparisons *post hoc* test; Arc Aβ + Vehicle: 12.67 (11.42-15.51) s vs Arc Aβ + XBD: 9.542 (8.643-12.67) s, p=0.44) and accuracy comparison (Kruskal–Wallis test with a Dunn's multiple comparisons *post hoc* test; Arc Aβ + Vehicle: 33.33 (16.66-50) % vs Arc Aβ + XBD: 58.33 (45.83-83.33) %, p=0.19) between the different treatment groups in the Retest 2 phase. Data are represented as median with their respective interquartile range. $*p < 0.05$. ns: not significant.

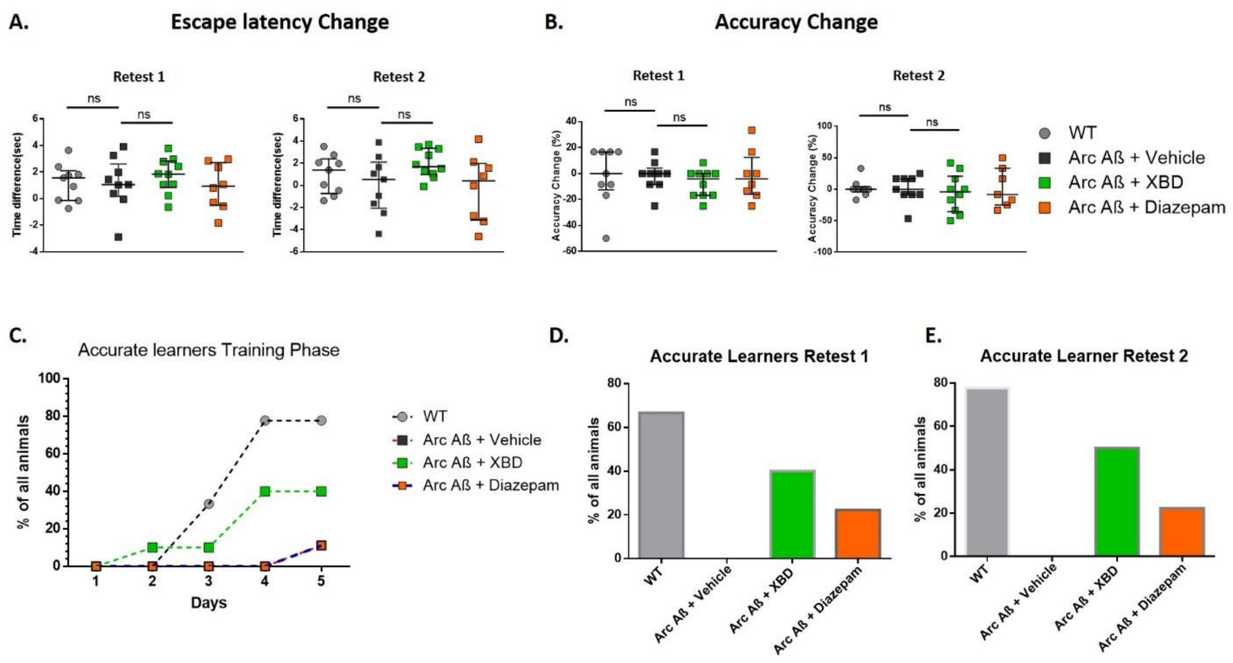

Supplementary Figure 8: A. Escape latency change in Retest 1 and 2 from the training phase for the different chronic treatment groups. WT (n=9), Arc Aβ + Vehicle (n=9), Arc Aβ + XBD (n=10) and Arc Aβ + Diazepam (n=8). B. Accuracy change in Retest 1 and 2 from the training phase for the different chronic treatment groups. WT (n=9), Arc Aβ + Vehicle (n=9), Arc Aβ + XBD (n=10) and Arc Aβ + Diazepam (n=8). C. Accurate learners during the 5-day training phase for different treatment groups. D. Bar plot showing Accurate learners in Retest 1. E. Bar plot showing Accurate learners in Retest 2. Data are represented as median with their respective interquartile range. *$p < 0.05$. ns: not significant.

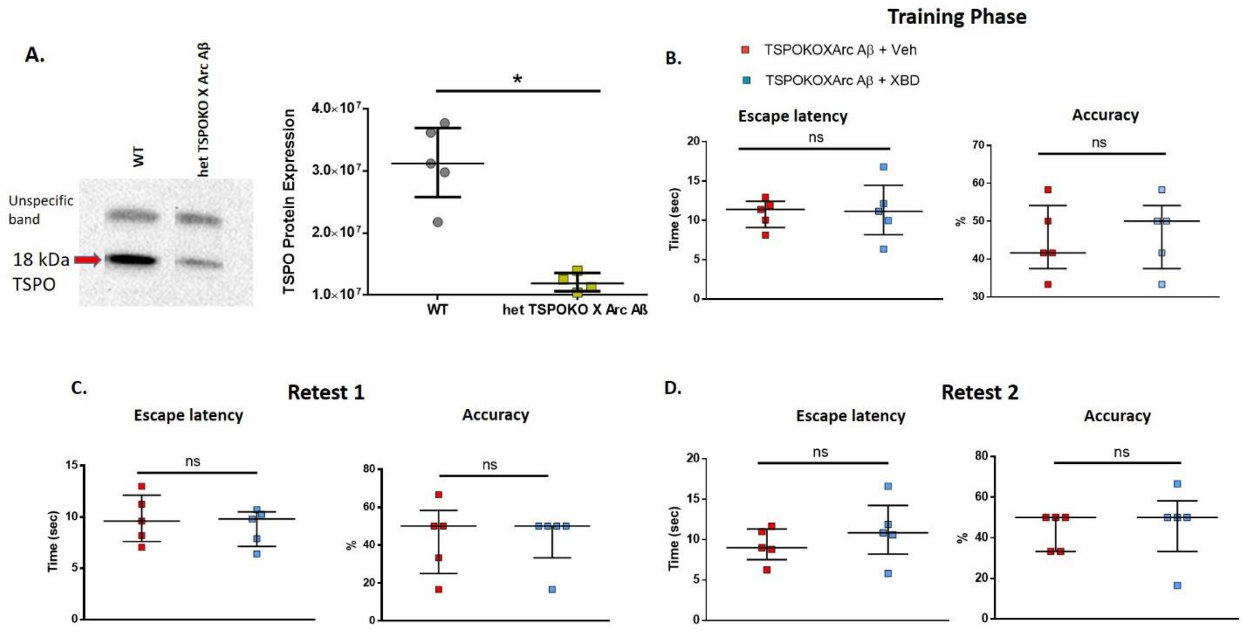

Supplementary Figure 9: TSPO protein is responsible for XBD173-mediated spatial learning improvement. A. TSPO protein expression in WT vs hetTSPOKO X Arc Aβ mice. The blot image shows an additional unspecific band in addition to the 18 kDa TSPO protein. The TSPO protein expression is normalized in reference to the total protein content. (Mann–Whitney U test: WT: 3.120e+007 (2.578e+007 - 3.692e+007) vs hetTSPOKO X Arc Aβ: 1.189e+007 (1.064e+007 - 1.357e+007) ; p=0.0159) B. Escape latency (Mann–Whitney U test: hetTSPOKO X Arc Aβ + XBD: 11.16 (8.168-14.46) s vs hetTSPOKO X Arc Aβ + Veh: 11.40 (9.097-12.43) s, p=0.944) and accuracy comparison (Mann–Whitney U test: hetTSPOKO X Arc Aβ + XBD: 50.00 (37.50-54.17) % vs hetTSPOKO X Arc Aβ + Veh (n=5): 41.67 (37.50-54.17) %, p=0.904) between the different treatment groups in the training phase. hetTSPOKO X Arc Aβ + Veh (n=5), and hetTSPOKO X Arc Aβ + XBD (n=5) groups. C. Escape latency (Mann–Whitney U test: hetTSPOKO X Arc Aβ + XBD: 9.800 (7.138-10.50) s vs hetTSPOKO X Arc Aβ + Veh: 9.600 (7.62-12.13) s, p=0.66) and accuracy comparison (Mann–Whitney U test: hetTSPOKO X Arc Aβ + XBD: 50.00 (33.33-50) % vs hetTSPOKO X Arc Aβ + Veh: 50.00 (25.00-58.33) %, p>0.99) between the different treatment groups in the Retest 1 phase. D. Escape latency (Mann–Whitney U test: hetTSPOKO X Arc Aβ + XBD: 10.86 (8.2-14.24) s vs hetTSPOKO X Arc Aβ + Veh: 9 (7.528-11.33) s, p=0.53) and accuracy comparison (Mann–Whitney U test: hetTSPOKO X Arc Aβ + XBD: 50.00 (33.33-58.33) % vs hetTSPOKO X Arc Aβ + Veh: 50 (33.33-50) %, p=0.64) between the different treatment groups in the Retest 2 phase. Data are represented as median with their respective interquartile range. $*p < 0.05$. ns: not significant. ** For western blot quantification of TSPO, protein concentration was adjusted to 2 µg/µl using a loading buffer (LDS Sample Buffer). Heating at 95°C for 5 minutes denatured the proteins before loading them onto 10% SDS gels. Gel electrophoresis was performed, initially at 100 V for 20 minutes and then at 200 V to separate proteins by size. Images were captured with a BioRad Chemi Doc XRS+ Molecular Imager. For blotting, proteins were transferred to a polyvinylidene fluoride membrane using a tank blot system with 80 V for 60 minutes. Stain-free blot images were taken for protein quantification. After blocking, the primary antibody (Recombinant anti-PBR antibody [EPR5384] (ab109497)) was added and incubated overnight. The membrane was washed, incubated with a secondary antibody, washed again, and

exposed to Enhanced Chemiluminescence reagent, and images were analyzed with ImageLab software. We used total protein as a reference for normalization

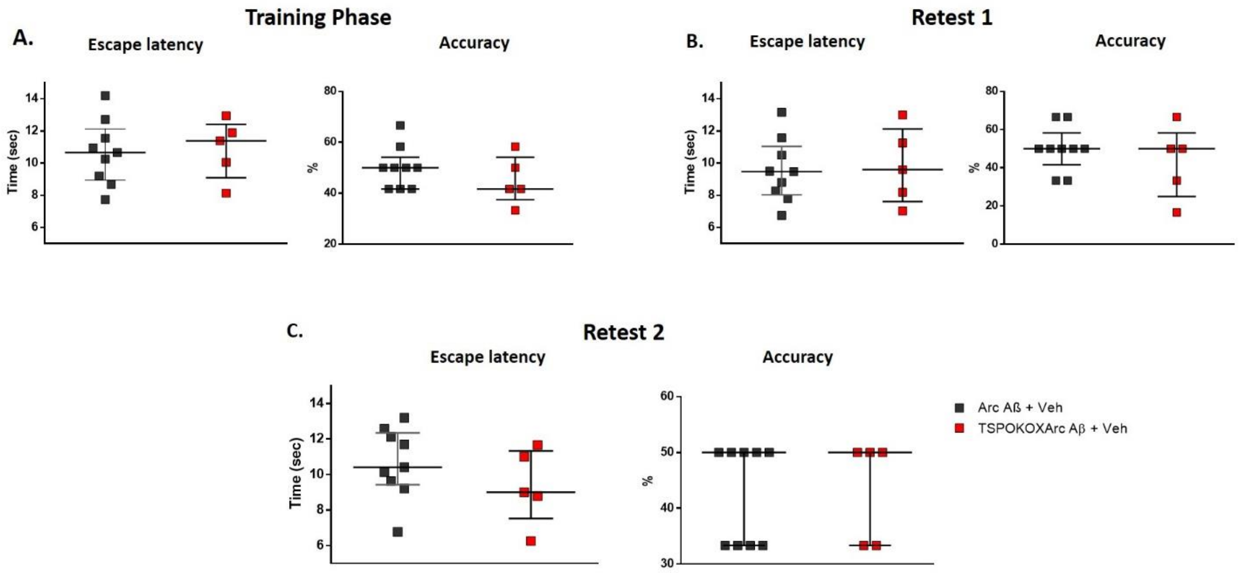

Supplementary Figure 10: A. Escape latency and accuracy comparison between the hetTSPOKO X Arc Aβ + Veh (n=5) and Arc Aβ + Veh (n=9) in the training phase. B. Escape latency and accuracy comparison between the hetTSPOKO X Arc Aβ + Veh and Arc Aβ + Veh in the Retest 1. C. Escape latency and accuracy comparison between the hetTSPOKO X Arc Aβ + Veh and Arc Aβ + Veh in the Retest 2.

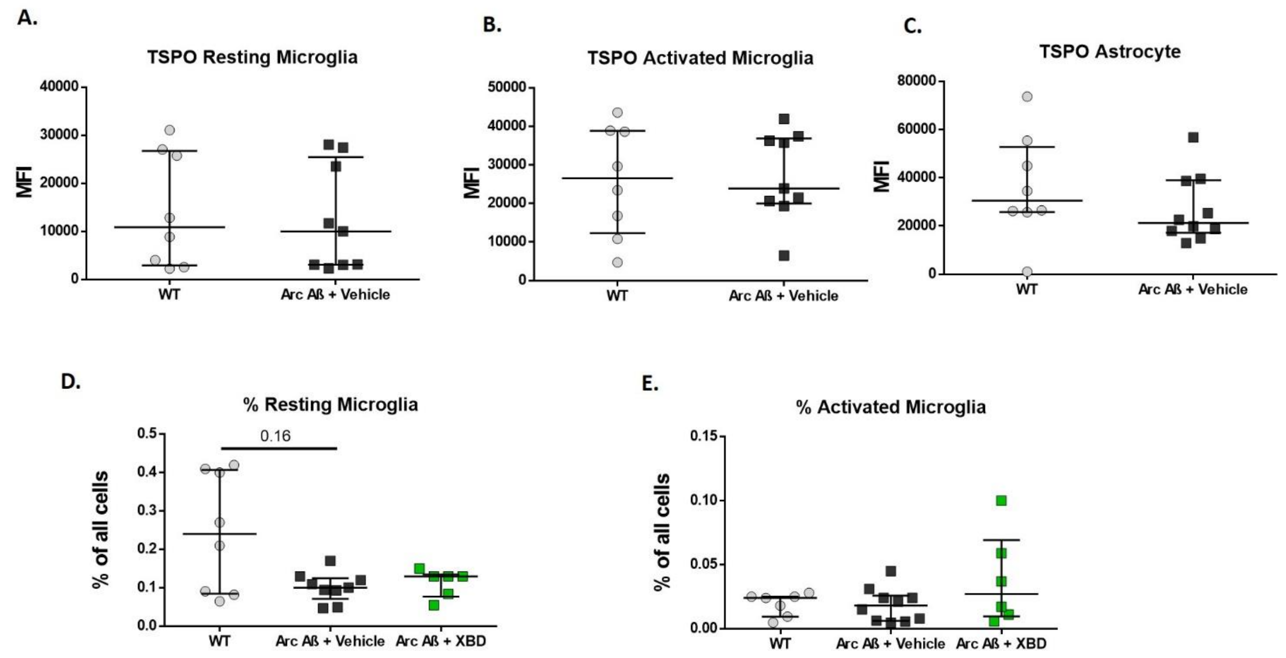

Supplementary Figure 11: A. Resting Microglial TSPO MFI comparison between WT (n=8) and Arc Aβ + Veh (n=9). (Mann–Whitney U test: WT: 10835 (2907-26707) vs Arc Aβ + Veh: 9996 (3037-25446), p=0.8689) B. Activated Microglial TSPO MFI comparison between WT (n=8) and Arc Aβ + Veh (n=9). (Mann–Whitney U test: WT: 26543(12299-38840) vs Arc Aβ + Veh: 23876 (20007-36861), p>0.9999) C. Astrocyte TSPO MFI comparison between WT (n=8) and Arc Aβ + Veh (n=10). (Mann–Whitney U test: WT: 30545 (25841-52808) vs Arc Aβ + Veh: 21228 (17165 -38936), p=0.1720). D. % Resting microglia comparison across the groups WT (n=8), Arc Aβ + Veh (n=9) and Arc Aβ + XBD (n=6) (Kruskal–Wallis test with a Dunn's multiple comparisons *post hoc* test; WT: 0.2400 (0.08425-0.4075) % vs Arc Aβ + Veh: 0.1000 (0.0710-0.1250) %; p=0.16; Arc Aβ + Veh vs Arc Aβ + XBD: 0.1300 (0.07675-0.1350) % of all cells, p>0.9999). E. % Activated microglia comparison across the groups WT (n=7), Arc Aβ + Veh (n=10), and Arc Aβ + XBD (n=6) Data are represented as median with their respective interquartile range. *p < 0.05. ns: not significant. **n sample size is pooled from both cortex and hippocampus.

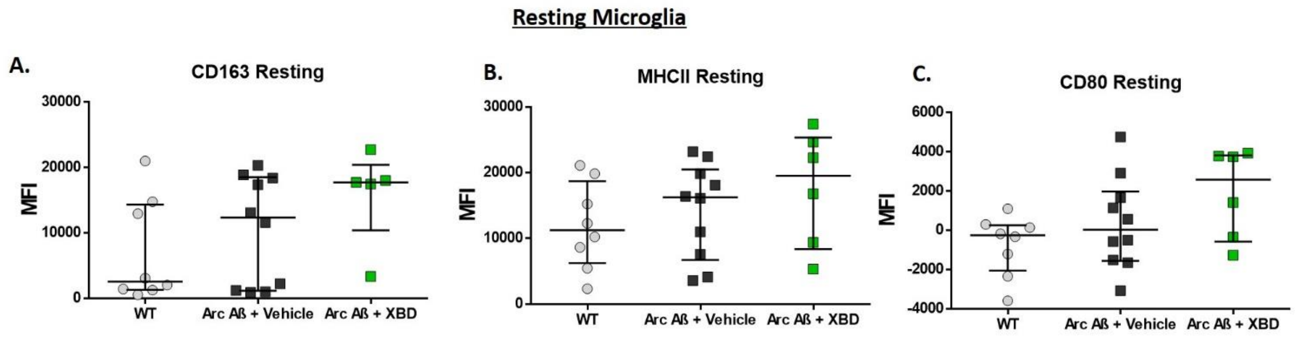

Supplementary Figure 12: A. Resting Microglial CD163 MFI comparison across the chronic treated groups. WT (n=7), Arc Aβ + Veh (n=10) and Arc Aβ + XBD (n=5). B. Resting Microglial MHCII MFI comparison across the chronically treated groups. WT (n=8), Arc Aβ + Veh (n=10) and Arc Aβ + XBD (n=6). C. Resting Microglial CD80 MFI comparison across the chronic treated groups. WT (n=8), Arc Aβ + Veh (n=10) and Arc Aβ + XBD (n=6). *n sample size is pooled from both cortex and hippocampus

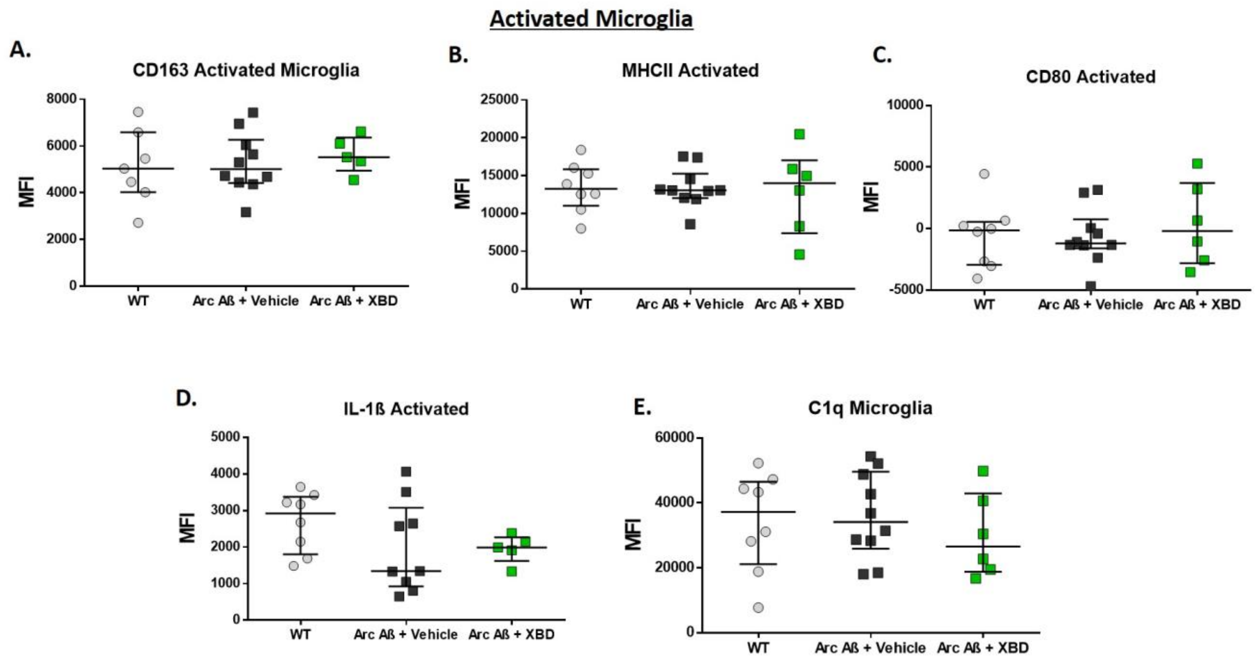

Supplementary Figure 13: A. Activated Microglial CD163 MFI comparison across the chronic treated groups. WT (n=7), Arc Aβ + Veh (n=10) and Arc Aβ + XBD (n=5). B. Activated Microglial MHCII MFI comparison across the chronic treated groups. WT (n=8), Arc Aβ + Veh (n=10) and Arc Aβ + XBD (n=6). C. Activated Microglial CD80 MFI comparison across the chronic treated groups. WT (n=7), Arc Aβ + Veh (n=10) and Arc Aβ + XBD (n=6). D. Activated Microglial IL-1β MFI comparison across the chronic treated groups. WT (n=7), Arc Aβ + Veh (n=9) and Arc Aβ + XBD (n=6). E. Activated Microglial C1q MFI comparison across the chronic treated groups. WT (n=7), Arc Aβ + Veh (n=10) and Arc Aβ + XBD (n=6). *n sample size is pooled from both cortex and hippocampus

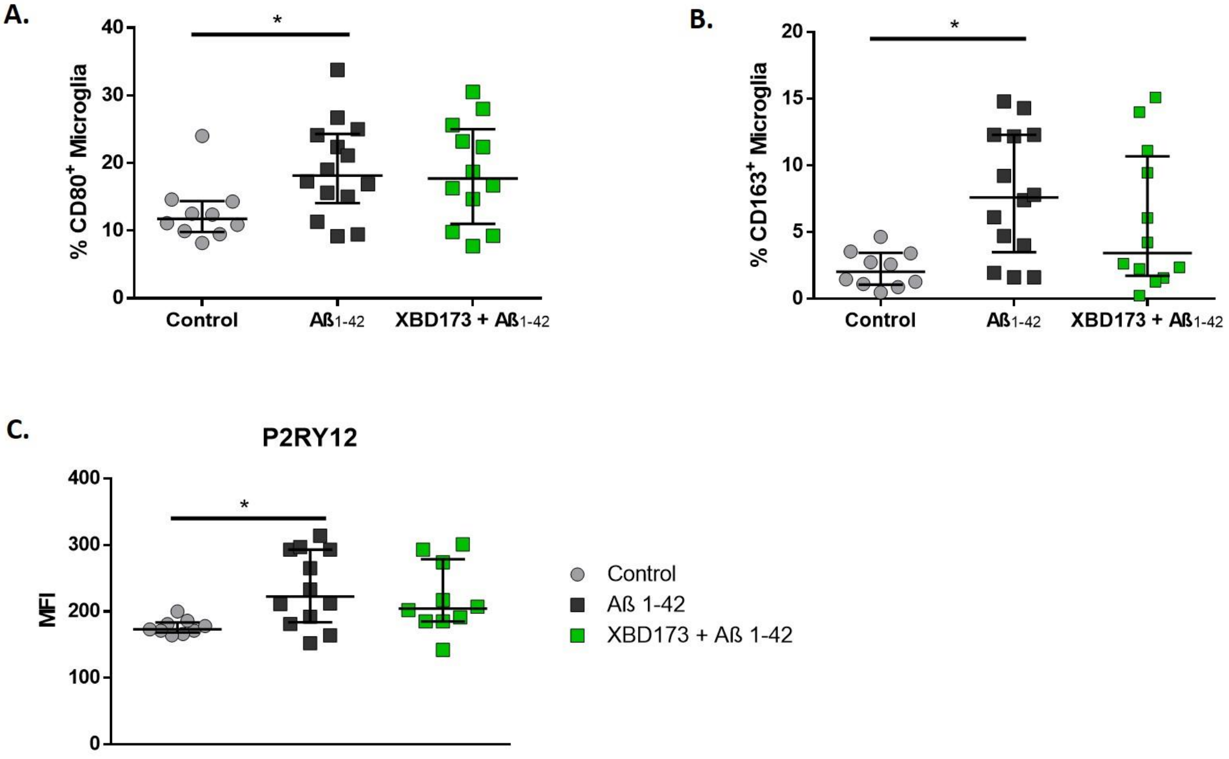

Supplementary Figure 14: Ex vivo XBD173 treatment doesn't affect the CD80 and CD163 responsive microglia which are altered via $A\beta_{1-42}$ oligomers. A. % of CD80 responsive microglia in the cortex and hippocampus in different treatment groups. Control (n=9), $A\beta_{1-42}$ (n=12) and XBD173 + $A\beta_{1-42}$ (n=10) (Kruskal–Wallis test with a Dunn's multiple comparisons *post hoc* test; Control: 11.9 (10.35-13.75) % vs $A\beta_{1-42}$: 20.55 (17.08-26.4) %, p=0.0013; $A\beta_{1-42}$ vs XBD173 + $A\beta_{1-42}$: 17.70 (10.35-23.68) %, p=0.3081). B. % of CD163 responsive microglia in the cortex and hippocampus in the different treatment groups. Control (n=9), $A\beta_{1-42}$ (n=12) and XBD173 + $A\beta_{1-42}$ (n=10) (Kruskal–Wallis test with a Dunn's multiple comparisons *post hoc* test; Control: 1.660 (1.155-3.495) % vs $A\beta_{1-42}$: 8.110 (4.55-13.38) %, p=0.0068; $A\beta_{1-42}$ vs XBD173 + $A\beta_{1-42}$: 2.705 (1.740-12.78) %, p=0.3662). C. Median fluorescence intensities (MFI) from FACS measurement for P2RY12 (Kruskal–Wallis test with a Dunn's multiple comparisons *post hoc* test; Control: 173 (168.5-183.5) vs $A\beta_{1-42}$: 222.5 (183.8-293), p=0.0162; $A\beta_{1-42}$ vs XBD173 + $A\beta_{1-42}$: 204.5 (185-278.8), p>0.99) for different treatment treatment groups. Data are represented as median with their respective interquartile range. *$p < 0.05$. ns: not significant.

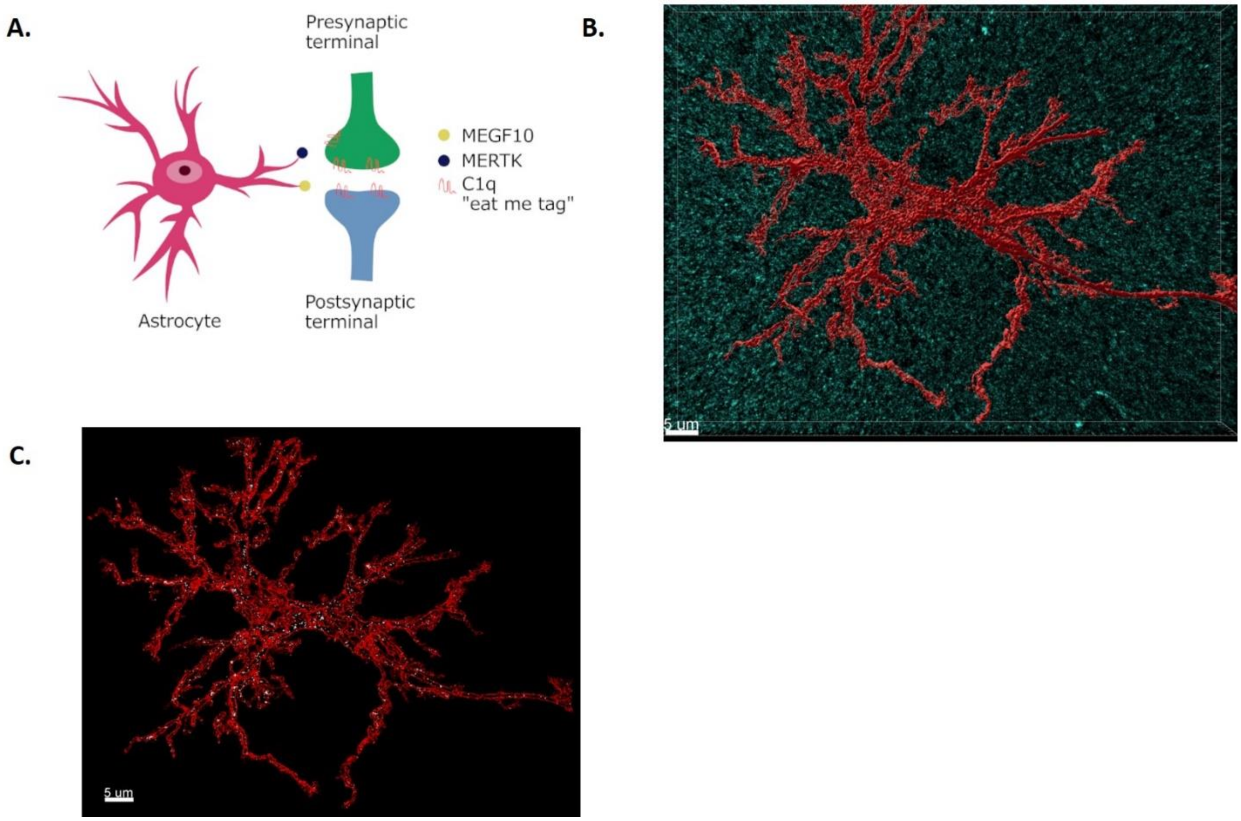

Supplementary Figure 15: A. Schematic showing astrocytic receptors (MEGF10 and MERTK) in direct elimination of synapses via C1q "eat-me tag". B. Individual high-resolution reconstructed astrocyte (shown in red) and C1q (shown in Cyan). C. Rendered outline of astrocyte (red) with colocalization between astrocytes and C1q marked in white. *Scale bar: 5 um*

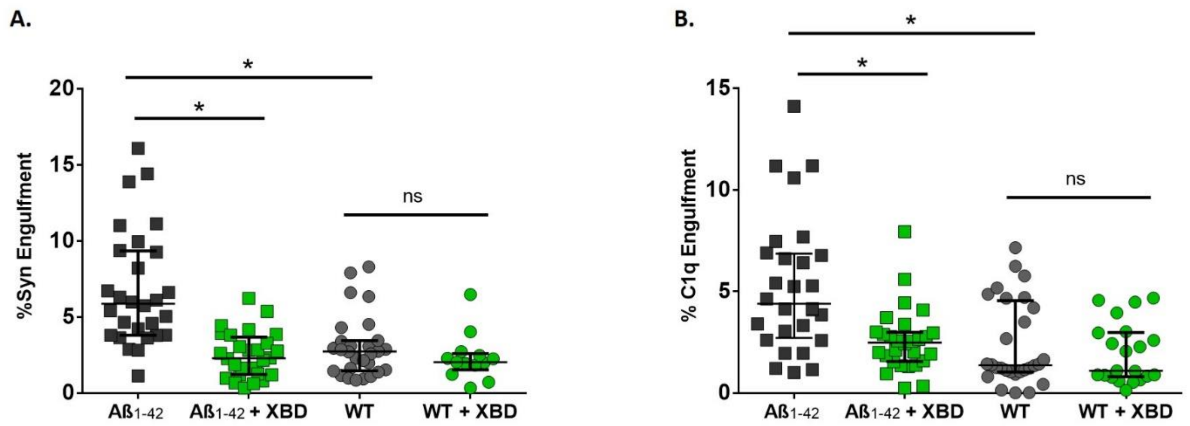

Supplementary Figure 16: Ex vivo incubation of slices with XBD173 reduces the increased astrocytic engulfment of Synaptophysin and C1q resulting from Aβ_{1-42} A. % of Syn engulfment by astrocyte quantified in different treatment groups in the hippocampus (Kruskal–Wallis test with a Dunn's multiple comparisons *post hoc* test; WT: 2.735 (1.470-3.448) % vs Aβ_{1-42}: 5.865 (3.803-9.345) %, $p<0.0001$; Aβ_{1-42} + XBD: 2.290 (1.233-3.678) % vs Aβ_{1-42}, $p<0.0001$; WT vs WT + XBD: 2.040 (1.530-2.605)%, $p=0.9346$); n=13-28 astrocytes collected from 4-8 mice per group). B. % of C1q engulfment by astrocyte quantified in different treatment groups in the hippocampus (Kruskal–Wallis test with a Dunn's multiple comparisons *post hoc* test; WT: 1.375 (1.043-4.550) % vs Aβ_{1-42}: 4.400 (2.723-6.870) %, $p=0.0003$; Aβ_{1-42} + XBD: 2.490 (1.560-2.998) % vs Aβ_{1-42}, $p=0.0077$; WT vs WT + XBD: 1.100 (0.810- 2.990) %, $p>0.9999$); n=21-28 astrocytes collected from 4-8 mice per group). Data are represented as median with their respective interquartile range. *$p < 0.05$. ns: not significant.
